# Effects of Aging on Glucose and Lipid Metabolism in Mice

**DOI:** 10.1111/acel.14462

**Published:** 2024-12-27

**Authors:** Evan C. Lien, Ngoc Vu, Anna M. Westermark, Laura V. Danai, Allison N. Lau, Yetiş Gültekin, Matthew A. Kukurugya, Bryson D. Bennett, Matthew G. Vander Heiden

**Affiliations:** ^1^ Department of Metabolism and Nutritional Programming Van Andel Institute Grand Rapids Michigan USA; ^2^ Koch Institute for Integrative Cancer Research Massachusetts Institute of Technology Cambridge Massachusetts USA; ^3^ Calico Life Sciences LLC South San Francisco California USA; ^4^ Department of Biochemistry and Molecular Biology University of Massachusetts Amherst Amherst Massachusetts USA; ^5^ Department of Medical Oncology Dana‐Farber Cancer Institute Boston Massachusetts USA; ^6^ Department of Biology Massachusetts Institute of Technology Cambridge Massachusetts USA

**Keywords:** aging, metabolic rate, mice, NAD

## Abstract

Aging is accompanied by multiple molecular changes that contribute to aging associated pathologies, such as accumulation of cellular damage and mitochondrial dysfunction. Tissue metabolism can also change with age, in part, because mitochondria are central to cellular metabolism. Moreover, the cofactor NAD^+^, which is reported to decline across multiple tissues during aging, plays a central role in metabolic pathways such as glycolysis, the tricarboxylic acid cycle, and the oxidative synthesis of nucleotides, amino acids, and lipids. To further characterize how tissue metabolism changes with age, we intravenously infused [U‐^13^C]‐glucose into young and old C57BL/6J, WSB/EiJ, and diversity outbred mice to trace glucose fate into downstream metabolites within plasma, liver, gastrocnemius muscle, and brain tissues. We found that glucose incorporation into central carbon and amino acid metabolism was robust during healthy aging across these different strains of mice. We also observed that levels of NAD^+^, NADH, and the NAD^+^/NADH ratio were unchanged in these tissues with healthy aging. However, aging tissues, particularly brain, exhibited evidence of upregulated fatty acid and sphingolipid metabolism reactions that regenerate NAD^+^ from NADH. These data suggest that NAD^+^‐generating lipid metabolism reactions may help to maintain the NAD^+^/NADH ratio during healthy aging.

## Graphical abstract

Lien et al. report a characterization of metabolism with aging across three different strains of mice. They find that glucose metabolism is robust during healthy aging; however, aging tissues exhibit evidence of upregulated fatty acid and sphingolipid metabolism reactions that regenerate NAD^+^ from NADH.

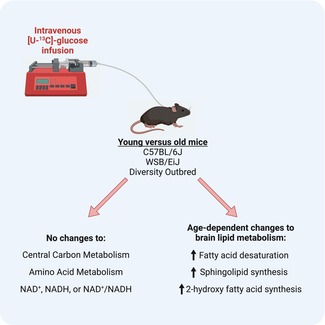

## 1 Introduction

Aging is a time‐dependent functional decline associated with increased susceptibility to pathology including diabetes, obesity, neurodegenerative diseases, cardiovascular diseases, and cancer. Several molecular hallmarks of aging have been characterized, including accumulation of cellular damage that contributes to tissue and organismal aging (López‐Otín et al. 2013). One well‐described hallmark is a progressive decline in mitochondrial function as organisms age with decreased mitochondrial electron transport chain function to support cellular respiration and ATP generation (Amorim et al. 2022; Green, Galluzzi, and Kroemer 2011; López‐Otín et al. 2013). Mitochondrial respiration also enables regeneration of the cofactor NAD^+^ (Luengo et al. 2021), and NAD^+^ has emerged as a critical molecule implicated in aging. Both NAD^+^‐producing reactions (e.g., de novo synthesis from tryptophan, nicotinamide salvage) and NAD^+^‐consuming reactions (e.g., sirtuins, poly(ADP‐ribose) polymerases, CD38 NADase) have been reported to be dysregulated with age, leading to a decline in NAD^+^ levels with age in many tissues in multiple organisms (McReynolds, Chellappa, and Baur 2020; Covarrubias et al. 2021; Camacho‐Pereira et al. 2016). Based on these observations, administration of NAD^+^ precursors such as nicotinamide riboside and nicotinamide mononucleotide is being explored as a potential therapy to extend longevity and health span (Fang et al. 2016; Mills et al. 2016).

The proposed mechanisms downstream of NAD^+^ depletion that contribute to aging are largely focused around the role of NAD^+^ as a cosubstrate for enzymes such as sirtuins and poly(ADP‐ribose) polymerases (Verdin 2015). However, NAD^+^ also plays a central role in cell metabolism, serving as an essential cofactor for redox reactions involved in glycolysis, the tricarboxylic acid (TCA) cycle, and the oxidative synthesis of macromolecules such as nucleotides, amino acids, and lipids (Bao et al. 2016; Birsoy et al. 2015; Diehl et al. 2019; Li et al. 2022; Sullivan et al. 2015; Titov et al. 2016; Vander Heiden and DeBerardinis 2017). Therefore, both aging associated mitochondrial dysfunction and a decline in NAD^+^ levels might be expected to alter cellular metabolism in ways that may contribute to the aging process. Indeed, several studies have characterized how various metabolic processes change with age (Anderson and Weindruch 2010; Gomes et al. 2013; Goyal et al. 2017; Hertel et al. 2016; Houtkooper et al. 2011; Laye et al. 2015; López‐Otín et al. 2016; Ross et al. 2010; Walters et al. 2018).

One approach for characterizing metabolism in vivo is using intravenous infusion of stable isotope‐labeled nutrients to trace the fate of infused nutrients into downstream metabolites within tissues by mass spectrometry (MS) (Bartman et al. 2023; Bartman, TeSlaa, and Rabinowitz 2021). This technique has been used to better understand nutrient utilization by normal tissues and tumors (Faubert et al. 2021; Hui et al. 2020, 2017). Here, we infused [U‐^13^C]‐glucose into healthy young and old mice to explore how tissue glucose utilization changes during aging. We examined liver, gastrocnemius muscle, and brain tissues across three different mouse strains: C57BL/6J mice, the longer lived WSB/EiJ in‐bred mice (Yuan et al. 2009), and genetically heterogeneous diversity outbred (DO) mice. Surprisingly, despite distinct metabolic features associated with these mouse strains, such as different sensitivities to high‐fat diet‐induced obesity and differences in insulin sensitivity (Lee et al. 2011; Bachmann et al. 2022), we found that glucose incorporation into central carbon and amino acid metabolism was robust across these strains of aging mice. Moreover, we did not measure significant declines in the levels of NAD^+^, NADH, or a change in NAD^+^/NADH ratio across the tissues analyzed. Rather, we observed higher levels of unsaturated fatty acids and sphingolipids in aged tissues, particularly the brain, that are produced via fatty acid desaturation and sphingolipid synthesis reactions that regenerate NAD^+^ from NADH. Together, these data provide a resource for how glucose utilization and lipid metabolism are altered with aging in three distinct mouse strains.

## 2 Results

### 2.1 Determining an Optimal [U‐ ^13^C]‐Glucose Infusion Rate to Trace Glucose Fate in Tissues in Aging Mice

**FIGURE 1:**
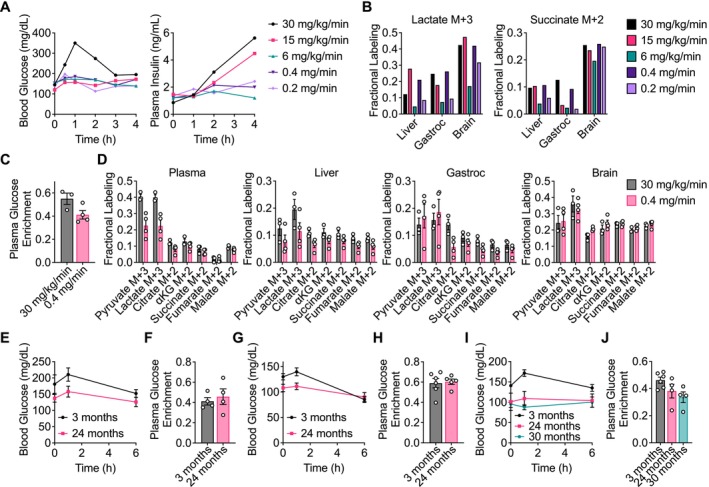
Determining an optimal [U‐^13^C]‐glucose intravenous infusion rate in aging mice. (A) Serial sampling of blood glucose and plasma insulin levels over time in C57BL/6J mice infused with [U‐^13^C]‐glucose with the indicated infusion rates. *n* = 1 mouse per infusion rate. (B) Fractional labeling of [M + 3] lactate and [M + 2] succinate in liver, gastrocnemius muscle (gastroc), and brain tissues from C57BL/6J mice infused with [U‐^13^C]‐glucose with the indicated infusion rates for 4 h. *n* = 1 mouse per infusion rate. Plasma glucose enrichment (C) and fractional labeling of the indicated metabolite isotopomers in plasma, liver, gastroc, and brain tissues (D) from C57BL/6J mice infused with [U‐^13^C]‐glucose at 30 mg/kg/min for 4 h (*n* = 3) or 0.4 mg/min for 6 h (*n* = 4). Serial sampling of blood glucose levels over time (E) and plasma glucose enrichment (F) from 3‐month‐old (*n* = 4) versus 24‐month‐old (*n* = 4) C57BL/6J mice infused with [U‐^13^C]‐glucose at 0.4 mg/min for 6 h. Serial sampling of blood glucose levels over time (G) and plasma glucose enrichment (H) from 3‐month‐old (*n* = 6) versus 24‐month‐old (*n* = 5) WSB/EiJ mice infused with [U‐^13^C]‐glucose at 0.4 mg/min for 6 h. Serial sampling of blood glucose levels over time (I) and plasma glucose enrichment (J) from 3‐month‐old (*n* = 6), 24‐month‐old (*n* = 4), and 30‐month‐old (*n* = 4) DO mice infused with [U‐^13^C]‐glucose at 0.4 mg/min for 6 h. Data are presented as mean ± SEM.

To study how glucose fate changes in tissues in aging mice, we first determined an optimal [U‐^13^C]‐glucose infusion rate that would allow tissue metabolite labeling comparisons between young and old mice. Because older animals tend to develop insulin resistance, which impacts whole‐body metabolism (Ehrhardt et al. 2019; Reynolds et al. 2019), we wanted to avoid examining changes in glucose metabolism that result from differences in insulin signaling responses between young and old mice. Therefore, we sought a glucose infusion protocol that minimally impacts blood glucose and insulin levels, while still labeling sufficient metabolites in tissues to permit analysis. In a previous study evaluating the contribution of glucose to the metabolism of lung adenocarcinoma tumors in vivo, we found that a glucose infusion rate of 30 mg/kg/min allowed substantial labeling of tumor metabolites (Davidson et al. 2016). Using this rate as a starting point, we also tested rates of 15 mg/kg/min and 6 mg/kg/min (Figure 1A). For these infusion studies, an ~25 g C57BL/6J mouse was infused with 500 mg/mL, 250 mg/mL, or 100 mg/mL [U‐^13^C]‐glucose at a rate of 1.5 μL/min over 4 h to achieve final infusion rates of 30 mg/kg/min, 15 mg/kg/min, or 6 mg/kg/min, respectively, which ensured that each mouse was infused with the same total volume of glucose (Table S1). We found that only the 30 mg/kg/min infusion rate increased blood glucose levels, whereas both the 30 mg/kg/min and 15 mg/kg/min infusion rates raised plasma insulin levels (Figure 1A). This suggested that 6 mg/kg/min may be an appropriate infusion rate; however, this led to substantially reduced labeling of metabolites from [U‐^13^C]‐glucose in the tissues analyzed (Figure 1B).

We next speculated that perhaps infusing a more concentrated [U‐^13^C]‐glucose solution at a slower rate might improve downstream metabolite labeling and have less of an effect on blood glucose and insulin. To this end, we tested two “fixed volume” infusions that were not adjusted for animal body weight, in which 200 μL of a 500 mg/mL or a 250 mg/mL [U‐^13^C]‐glucose solution were delivered into C57BL/6J mice over 4 h (an infusion pump rate of 0.8 μL/min), resulting in final infusion rates of 0.4 mg/min or 0.2 mg/min, respectively (Table S1). We found that both of these rates slightly elevated blood glucose and insulin, but not to the same extent as the 30 mg/kg/min and 15 mg/kg/min rates (Figure 1A), and the 0.4 mg/min infusion rate led to downstream metabolite labeling that was comparable to the 30 mg/kg/min infusion rate (Figure 1B).

We next confirmed in a larger cohort of mice that an infusion rate of 0.4 mg/min is comparable to our previously used rate of 30 mg/kg/min to allow sufficient tissue labeling for analysis. Both infusion rates resulted in substantial plasma [U‐^13^C]‐glucose enrichment, with 0.4 mg/min leading to only slightly lower enrichment in plasma glucose than 30 mg/kg/min (Figure 1C). Notably, even though the 0.4 mg/min rate was not adjusted for the body weights of different mice, there was low variability in plasma glucose enrichment across different mice (Figure 1C). Labeling of downstream metabolites including pyruvate, lactate, and TCA cycle intermediates in plasma, liver, gastrocnemius, and brain tissues was also comparable between these infusion rates (Figure 1D). Therefore, an infusion rate of 0.4 mg/min was determined to label metabolites to sufficient levels in tissues for analysis.

Since different tissues may have different nutrient uptake and exchange rates, achieving steady‐state labeling is necessary for making valid comparisons between different tissues, and would allow us to evaluate whether aging alters the direct and indirect contributions of glucose to the pools of various downstream metabolites. To confirm that labeling reached steady state in blood and tissues, we infused C57BL/6J mice with [U‐^13^C]‐glucose for 0.5, 2, 4, and 6 h. Plasma [U‐^13^C]‐glucose enrichment reached steady state by 6 h of infusion (Figure S1A). Similarly, labeling of pyruvate, lactate, TCA cycle intermediates, and nonessential amino acids also reached steady state by 6 h in plasma, liver, gastrocnemius, and brain tissues (Figure S1B–D). Therefore, a [U‐^13^C]‐glucose infusion in mice at a rate of 0.4 mg/min for 6 h is able to achieve steady‐state labeling of these metabolites in mouse tissues.

**FIGURE 2:**
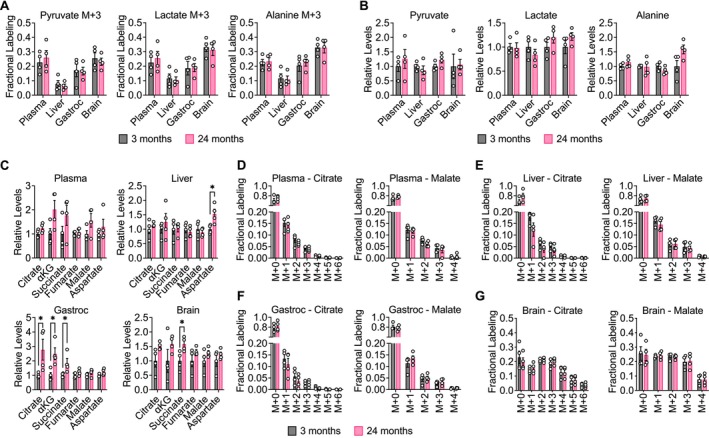
Glucose contribution to central carbon metabolism is robust in aging C57BL/6J mice. C57BL/6J mice, 3‐month‐old (*n* = 4) versus 24‐month‐old (*n* = 4), were infused with [U‐^13^C]‐glucose at 0.4 mg/min for 6 h. [M + 3] fractional labeling (A) and relative levels (B) of pyruvate, lactate, and alanine in the indicated tissues. (C) Relative levels of TCA cycle metabolites in the indicated tissues. Mass isotopomer distributions of citrate and malate in plasma (D), liver (E), gastrocnemius muscle (F), and brain (G) tissues. Data are presented as mean ± SEM. Relative metabolite levels (B, C) represent mass spectrometry peak areas that are normalized to an internal standard and tissue weight, before being normalized relative to the average value in 3‐month‐old mice. Comparisons were made using a two‐tailed Student's *t* test. *p < 0.05.

Finally, we assessed whether different [U‐^13^C]‐glucose infusion rates would impact metabolite labeling in tissues from young versus old C57BL/6J mice. In mice infused at a rate of 30 mg/kg/min, labeling of pyruvate and lactate in tissues, particularly gastrocnemius and brain, was higher in 24‐month‐old compared to 3‐month‐old mice (Figure S2A,B). Interestingly, this difference was no longer observed in mice infused at a rate of 0.4 mg/min (Figure 2A). These observations suggest that infusion rate differences may indeed alter tissue metabolite labeling patterns. While we acknowledge that many studies use body weight‐adjusted [U‐^13^C]‐glucose infusion rates, our data empirically found that a fixed volume infusion rate of 0.4 mg/min for 6 h minimized effects on blood glucose and insulin levels and reached steady‐state tissue labeling, enabling comparison of tissue metabolite labeling between young and old mice.

### 2.2 Glucose Contribution to Central Carbon Metabolism Is Robust in Aging Mice

We next conducted steady‐state [U‐^13^C]‐glucose infusions in young and old cohorts of three distinct mouse strains. For C57BL/6J and WSB/EiJ mice, 3‐month‐old versus 24‐month‐old mice were compared. For diversity outbred (DO) mice, 3‐month‐old, 24‐month‐old, and 30‐month‐old mice were compared. Body weights of all infused mice are provided in Table S2. Importantly, none of the older mice exhibited overt signs of disease, allowing us to evaluate how glucose metabolism changes with healthy aging. Mice were fasted for 4 h prior to infusions, and for all mouse strains, older mice had lower fasting blood glucose at the beginning of the infusion (Figure 1E,G,I). Similar to Figure 1A, the infusions only slightly raised blood glucose levels within the first hour, and blood glucose levels returned to near baseline levels by the end of the infusion, which was likely caused by the slight elevation in plasma insulin with the infusion (Figure 1E,G,I). Moreover, similar plasma [U‐^13^C]‐glucose enrichment was obtained between young and old mice for each mouse strain (Figure 1F,H,J), further supporting that a fixed volume infusion can achieve similar plasma glucose enrichment between mice with different body weights.

We first examined the contribution of glucose to the glycolytic products pyruvate and lactate in aging mice. We also considered labeling of alanine, which can be produced from pyruvate by transamination. Of note, because plasma glucose enrichment was similar between young and old mice (Figure 1F,H,J), we compared the fractional labeling of these downstream metabolites without normalization to plasma glucose enrichment. Normalizing tissue metabolite labeling to plasma glucose enrichment assumes that fractional labeling scales linearly with the amount of labeled glucose in the blood, which is not necessarily the case (e.g., see Figure 1C,D). Nevertheless, we also provide all metabolite labeling data normalized to plasma glucose enrichment in Tables S3–, S5. In C57BL/6J mice, labeling of [M + 3] pyruvate, lactate, and alanine was unchanged in plasma, liver, gastrocnemius, and brain tissues between young and old mice (Figure 2A). Total levels of these metabolites also did not change in an age‐dependent manner (Figure 2B). Similar results were observed in WSB/EiJ (Figure S3A–F) and DO mice (Figure S3G–L). These data suggest that the contribution of glucose carbon to glycolysis in plasma, liver, gastrocnemius, and brain tissues remains robust in mice during healthy aging.

We next evaluated the contribution of glucose carbon to the TCA cycle intermediates citrate, ⍺‐ketoglutarate (⍺KG), succinate, fumarate, and malate. We also considered labeling of aspartate, which can be produced from oxaloacetate by transamination. In C57BL/6J mice, total levels of these metabolites may slightly increase with age in some tissues, particularly in gastrocnemius muscle and brain (Figure 2C). These differences, however, were not observed in WSB/EiJ and DO mice. In WSB/EiJ mice, levels of TCA cycle intermediates decreased with age in some tissues, particularly in the brain (Figure S5A–D), whereas in DO mice, no robust age‐dependent changes were observed (Figure S6A–D). The lack of consistent age‐associated changes in these metabolites across all three mouse strains suggests that the differences observed do not represent a general feature of murine aging. In each mouse strain, examining all mass isotopologs of TCA cycle intermediates in plasma, liver, gastrocnemius, and brain tissues revealed no differences between young and old mice in the labeling of these metabolites (Figure 2D–G, Figures S4A–D, S5E–H, S6E–H). These results indicate that the contribution of glucose carbon to the TCA cycle in plasma, liver, gastrocnemius, and brain tissues remains robust in mice during healthy aging.

Despite the lack of age‐dependent changes, we observed several age‐independent differences in tissue TCA cycle metabolite labeling. In plasma, liver, and gastrocnemius, the most abundant mass isotopolog for most TCA cycle intermediates was the [M + 1] isotopolog (Figure 2D–F, Figures S4A–C, S5E–G, S6E–G). This in vivo labeling pattern is distinct from [U‐^13^C]‐glucose labeling of TCA cycle metabolites of cultured cells in vitro, in which the [M + 2] and [M + 3] isotopologs are typically most abundant (Davidson et al. 2016; Sellers et al. 2015). It is possible that these [M + 1] species may arise from either TCA cycling or carboxylation of TCA cycle metabolites from [U‐^13^C]‐glucose‐derived CO_2_ (Duan et al. 2022; Hensley et al. 2016). High [M + 1] labeling of TCA cycle intermediates from in vivo [U‐^13^C]‐glucose tracing has also been observed in previous studies (Davidson et al. 2016; Duan et al. 2022; Hensley et al. 2016). In contrast, labeling of TCA cycle metabolites in brain tissues was unique and more closely mirrored in vitro labeling of cultured cells with substantial [M + 2] and [M + 3] isotopologs (Figure 2G, Figures S4D, S5H, S6H). Moreover, a greater total fraction of TCA cycle metabolites in the brain were labeled by [U‐^13^C]‐glucose. This observation is consistent with the brain being a more glucose‐avid tissue (Hui et al. 2017).

### 2.3 Glucose Contribution to Amino Acid Metabolism Is Robust in Aging Mice

**FIGURE 3:**
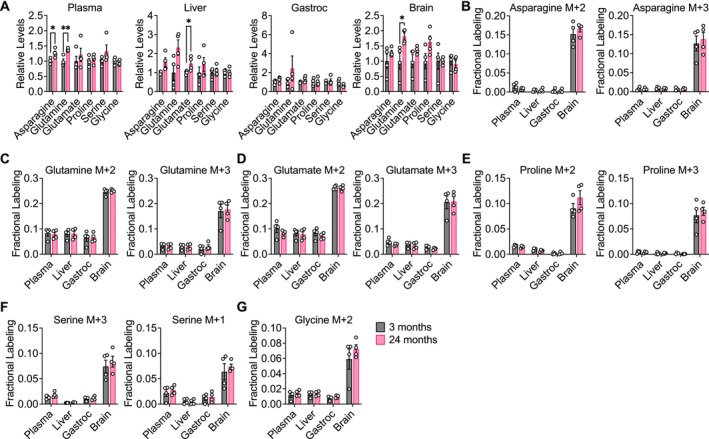
Glucose contribution to amino acid metabolism is robust in aging C57BL/6J mice. C57BL/6J mice, 3‐month‐old (*n* = 4) versus 24‐month‐old (*n* = 4), were infused with [U‐^13^C]‐glucose at 0.4 mg/min for 6 h. (A) Relative levels of the indicated amino acids in plasma, liver, gastrocnemius muscle, and brain tissues. Relative metabolite levels represent mass spectrometry peak areas that are normalized to an internal standard and tissue weight, before being normalized relative to the average value in 3‐month‐old mice. Fractional labeling of [M + 2] and [M + 3] asparagine (B), [M + 2] and [M + 3] glutamine (C), [M + 2] and [M + 3] glutamate (D), [M + 2] and [M + 3] proline (E), [M + 3] and [M + 1] serine (F), and [M + 2] glycine (G) in the indicated tissues. Data are presented as mean ± SEM. Comparisons were made using a two‐tailed Student's *t* test. *p < 0.05, **p < 0.01.

We next examined the contribution of glucose carbon to the nonessential amino acids asparagine, glutamine, glutamate, proline, serine, and glycine. In C57BL/6J mice, total levels of these metabolites did not robustly change with age, with some slight increases in liver and brain tissues of aged mice (Figure 3A). Similarly, consistent age‐dependent differences were not observed in WSB/EiJ and DO mice. Decreases in total levels of some amino acids were observed with age in some WSB/EiJ tissues, particularly in the gastrocnemius muscle and the brain (Figure S7A), whereas no robust age‐dependent changes were observed in DO mouse tissues (Figure S8A). In all mouse strains, labeling of these nonessential amino acids in plasma, liver, gastrocnemius, and brain tissues revealed no differences between young and old mice (Figure 3B–G, Figures S7B–G, S8B–G). These results indicate that the contribution of glucose carbon to nonessential amino acid metabolism is also robust in mice during healthy aging. Again, similar to labeling of the TCA cycle, tissue‐specific differences were observed—compared to plasma, liver, and gastrocnemius tissues, brain tissues exhibited a greater contribution of glucose carbon to nonessential amino acids in all mouse strains (Figure 3B–G, Figures S7B–G, S8B–G), which again may be consistent with the brain being a more glucose‐avid tissue (Hui et al. 2017).

### 2.4 Profiling of Polar Metabolites Does Not Suggest Major Age‐Dependent Changes in Metabolite Levels

Taken together, the earlier data suggest that glucose contribution to central carbon and amino acid metabolism is robust across healthy aging in mice and is consistent across mouse strains, including genetically diverse DO mice. Given no major age‐dependent differences in tissue metabolite labeling by [U‐^13^C]‐glucose, we also asked whether broader polar metabolite profiling by LC–MS would reveal any age‐dependent changes in tissue metabolite levels. We compared uninfused liver, gastrocnemius, and brain tissues from 3‐, 12‐, and 24‐month‐old C57BL/6J mice. Unsupervised hierarchical clustering of metabolomics data derived from analysis of these tissues did not result in clustering of the different tissues by age (Figure S9A–C), and principal component analyses further showed that the metabolomic profiles of liver, gastrocnemius, and brain tissues do not suggest major overall age‐dependent changes (Figure S9D–F).

### 2.5 Levels of NAD ^+^, NADH, and the NAD ^+^/NADH Ratio Are Stable in Mouse Tissues With Healthy Aging

**FIGURE 4:**
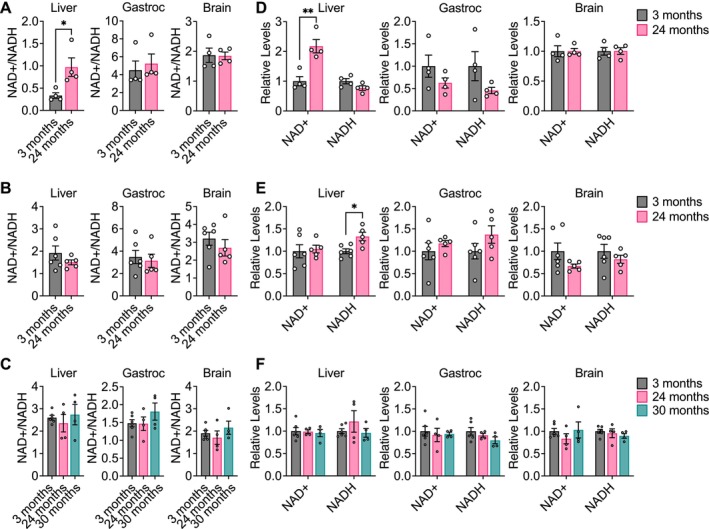
NAD^+^, NADH, and NAD^+^/NADH do not significantly change with healthy aging in mouse tissues. NAD^+^/NADH ratios in liver, gastrocnemius muscle, and brain tissues from young versus old C57BL/6J (A), WSB/EiJ (B), and DO (C) mice. Relative levels of NAD^+^ and NADH in in liver, gastrocnemius muscle, and brain tissues from young versus old C57BL/6J (D), WSB/EiJ (E), and DO (F) mice. C57BL/6J: 3 months *n* = 4, 24 months *n* = 4. WSB/EiJ: 3 months *n* = 6, 24 months *n* = 5. DO: 3 months *n* = 6, 24 months *n* = 4, 30 months *n* = 4. Data are presented as mean ± SEM. Relative levels (D–F) represent luminescence signals measured for either NAD^+^ or NADH from the NAD^+^/NADH‐Glo Assay Kit that are normalized to the average value in 3‐month‐old mice. Comparisons were made using a two‐tailed Student's *t* test. *p < 0.05, **p < 0.01.

Our data suggest that despite the mitochondrial dysfunction reported to occur during aging (Amorim et al. 2022; Green, Galluzzi, and Kroemer 2011; López‐Otín et al. 2013), the contribution of glucose carbon, either directly or indirectly, to glycolysis, the TCA cycle, and nonessential amino acids remains robust with healthy aging. Since these measurements do not provide direct information about metabolic flux through these pathways, this conclusion is not necessarily incompatible with aging associated impairment of mitochondrial function. Mitochondrial respiration is a major pathway for cells to regenerate NAD^+^, and impaired respiration might be expected to lower the NAD^+^/NADH ratio in cells, which would be consistent with reports that NAD^+^ declines with age in multiple organisms (McReynolds, Chellappa, and Baur 2020). Therefore, we evaluated the levels of NAD^+^, NADH, and the NAD^+^/NADH ratio in aging mouse tissues. To our surprise, we found that in all three strains of mice, the NAD^+^/NADH ratio was unchanged with age in liver, gastrocnemius, and brain tissues, with the exception of NAD^+^/NADH being slightly increased in liver from C57BL/6J mice (Figure 4A–C). Similarly, levels of NAD^+^ and NADH did not significantly change with age in all three mouse strains, with the exception of NAD^+^ being slightly increased in liver from C57BL/6J mice and NADH being slightly increased in liver from WSB/EiJ mice (Figure 4D–F). Impaired mitochondrial respiration typically results in increased fermentation of pyruvate to lactate, which serves as an alternative route to regenerate NAD^+^. However, since the contribution of glucose carbon to [M + 3] lactate in aging tissues was unchanged (Figure 2A, Figure S3B,H), we speculated that other metabolic pathways that regenerate NAD^+^ from NADH may be upregulated during healthy aging in mice to maintain the NAD^+^/NADH ratio and compensate for any decrease in mitochondrial function.

### 2.6 Fatty Acid Desaturation Increases in Aging Mouse Tissues

**FIGURE 5:**
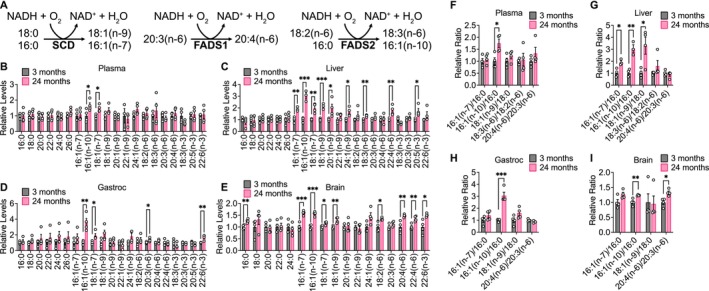
Fatty acid desaturation increases in aging C57BL/6J tissues. (A) Schematic of fatty acid desaturation reactions by SCD, FADS1, and FADS2. Relative levels of the indicated fatty acids in plasma (B), liver (C), gastrocnemius muscle (D), and brain (E) tissues from young versus old C57BL/6J mice. Relative fatty acid levels represent mass spectrometry peak areas that are normalized to an internal standard and tissue weight, before being normalized relative to the average value in 3‐month‐old mice. Relative fatty acid desaturation ratios in plasma (F), liver (G), gastrocnemius muscle (H), and brain (I) tissues from young versus old C57BL/6J mice. Relative fatty acid ratios are shown normalized to the average value in 3‐month‐old mice. 3 months *n* = 4, 24 months *n* = 4. Data are presented as mean ± SEM. Comparisons were made using a two‐tailed Student's *t* test. *p < 0.05, **p < 0.01, ***p < 0.001.

Increased membrane fatty acid desaturation in different species has been associated with decreased lifespan (Hulbert et al. 2007), and within the same species, membrane fatty acid desaturation increases with age in various tissues (Naudí et al. 2013). Moreover, caloric restriction, which robustly extends lifespan across multiple organisms, decreases membrane fatty acid desaturation in various mouse tissues (Jové et al. 2014; Lien et al. 2021; Miller et al. 2017). The production of unsaturated fatty acids is mediated by a family of fatty acid desaturase enzymes, including stearoyl‐CoA desaturase (SCD), fatty acid desaturase 1 (FADS1), and fatty acid desaturase 2 (FADS2), that introduce a double bond into various fatty acid substrates (Figure 5A). SCD produces the monounsaturated fatty acids 18:1(n‐9) and 16:1(n‐7) from 18:0 and 16:0, respectively. FADS1 produces polyunsaturated fatty acids, such as 20:4(n‐6) from 20:3(n‐6). FADS2 also primarily synthesizes polyunsaturated fatty acids, including 18:3(n‐6) from 18:2(n‐6), and has also been described to produce the monounsaturated fatty acid 16:1(n‐10) from 16:0 (Vriens et al. 2019). Notably, these fatty acid desaturases utilize NADH as a cofactor, regenerating NAD^+^ when introducing a double bond into fatty acids (Figure 5A). Upregulated production of unsaturated fatty acids may therefore maintain the NAD^+^/NADH ratio in tissues (Kim et al. 2019).

We examined the fatty acid composition of aging mouse tissues by derivatizing tissue fatty acids to fatty acid methyl esters (FAME) for analysis by gas chromatography–mass spectrometry (GC–MS). Indeed, we found that aging C57BL/6J mouse tissues, particularly brain and liver tissues, had higher levels of unsaturated fatty acids (Figure 5B–E). The ratios of the products of fatty acid desaturation reactions to their corresponding substrates have been used as surrogates for fatty acid desaturase activities (Lien et al. 2021; Vriens et al. 2019). Therefore, we assessed the 18:1(n‐9)/18:0 and 16:1(n‐7)/16:0 ratios as indicators of SCD activity, the 20:4(n‐6)/20:3(n‐6) ratio as a surrogate of FADS1 activity, and the 18:3(n‐6)/18:2(n‐6) and 16:1(n‐10)/16:0 ratios as surrogates of FADS2 activity. These ratios all tended to increase with age in C57BL/6J tissues, particularly in the liver and brain (Figure 5F–I). In WSB/EiJ mice, higher levels of several monounsaturated fatty acids were observed only in aged liver tissues, whereas plasma, gastrocnemius, and brain tissues exhibited few changes (Figure S10A–D). In DO mice, increased levels of unsaturated fatty acids were not observed in aging tissues, and in gastrocnemius tissues many fatty acid species decreased with age (Figure S11A–D). However, fatty acid desaturation ratios trended toward an increase in both aging WSB/EiJ and DO tissues (Figures S10E–H, S11E–H), with significant increases observed in the brain in all mouse strains analyzed. Taken together, these data indicate that increased fatty acid desaturation ratios are one of the most significant age‐dependent changes observed in our data sets collected across all three mouse strains, suggesting that fatty acid desaturation may increase with age in mice.

### 2.7 Aging Mouse Brain Tissue Exhibits Changes in Sphingolipid Metabolism

**FIGURE 6:**
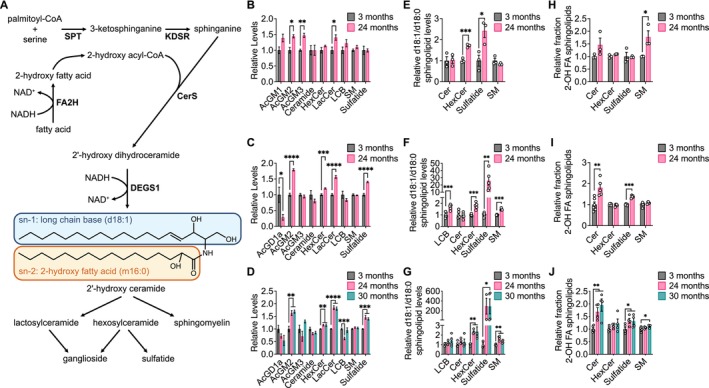
Aging brain tissues exhibit changes in sphingolipid metabolism. (A) Schematic of sphingolipid synthesis. Relative levels of the indicated sphingolipid classes in brain tissues from young versus old C57BL/6J (B), WSB/EiJ (C), and DO (D) mice. Relative d18:1/d18:0 ratios of sphingolipid levels of the indicated sphingolipid classes in brain tissues from young versus old C57BL/6J (E), WSB/EiJ (F), and DO (G) mice. Relative fraction of sphingolipids containing 2‐hydroxy fatty acids (2‐OH FA) within each indicated sphingolipid class in brain tissues from young versus old C57BL/6J (H), WSB/EiJ (I), and DO (J) mice. C57BL/6J: 3 months *n* = 3, 24 months *n* = 3. WSB/EiJ: 3 months *n* = 6, 24 months *n* = 5. DO: 3 months *n* = 6, 24 months *n* = 4, 30 months *n* = 4. Data are presented as mean ± SEM. Relative values represent raw data that are normalized relative to the average value in 3‐month‐old mice. Comparisons were made using a two‐tailed Student's *t* test. AcGM1, GM1 gangliosides; AcGM2, GM2 gangliosides; AcGM3, GM3 gangliosides; AcGD1a, GD1a gangliosides; HexCer, hexosylceramide; LacCer, lactosylceramide; LCB, long‐chain base; SM, sphingomyelin. *p < 0.05, **p < 0.01, ***p < 0.001, ****p < 0.0001.

We next conducted lipidomics analyses on aging brain tissues because increases in fatty acid desaturation ratios were observed most robustly in the brain. Strikingly, we found that brain tissues from older mice in all three mouse strains had higher levels of sphingolipids, including hexosylceramides (HexCer), lactosylceramides (LacCer), gangliosides, and sulfatides (Figure 6A–D). Upon considering the sphingolipid synthesis pathway, we noted that in a reaction analogous to the fatty acid desaturases, dihydroceramide desaturase (DEGS1) introduces a double bond into dihydroceramide to synthesize ceramide, which is coupled with the oxidation of NAD(P)H to NAD(P)^+^ (Figure 6A). When palmitoyl‐CoA is incorporated into sphingolipids by serine palmitoyltransferase (SPT), DEGS1 converts the d18:0 long‐chain base (LCB) in the sn‐1 position of dihydroceramide into a d18:1 LCB in the sn‐1 position of ceramide (Figure 6A). We found in all three mouse strains that aged brain tissues contained overall lower levels of d18:0‐containing sphingolipids and higher levels of d18:1‐containing sphingolipids (Figure S12A–C). By calculating the ratio of the total ion count of d18:1‐containing lipids versus the total ion count of d18:0‐containing lipids for each sphingolipid class, the d18:1/d18:0 ratios were found to be increased in aged brain tissues across all three mouse strains, particularly in HexCer and sulfatides (Figure 6E–G). In addition, we noted that d18:2‐containing sphingolipids can be generated by the addition of another double bond to the LCB of ceramide through the ceramide desaturase FADS3 (Karsai et al. 2020; Jojima et al. 2020), and overall levels of d18:2‐containing sphingolipids were also increased in aged brain tissues (Figure S12A–C). Collectively, these data suggest that sphingolipid desaturase activity, which is a route for NAD^+^ regeneration, may be higher in aged mouse brain tissue.

Finally, we also detected many sphingolipid species that contain a 2‐hydroxy fatty acid (2‐OH FA) at the sn‐2 position. 2‐OH FAs are synthesized by fatty acid 2‐hydroxylase (FA2H) in a reaction that is also coupled with the oxidation of NAD(P)H to NAD(P)^+^ (Figure 6A). In all three mouse strains, many 2‐OH FA‐containing sphingolipids were found to be elevated in the brains of older mice (Figure S13A–C). By calculating the ratio of the total ion count of 2‐OH FA‐containing lipids versus the total ion count of the corresponding nonhydroxylated FA‐containing lipids for each sphingolipid class, we found that the relative fraction of 2‐OH FA‐containing sphingolipids was increased in aged brain tissues, particularly in ceramides, sulfatides, and sphingomyelins (SM) (Figure 6H–J). These data suggest that the production of 2‐OH FA by FA2H, yet another potential route for NAD^+^ regeneration, may also be higher in aged mouse brain tissue.

## 3 Discussion

Aging is accompanied by alterations to biological processes that contribute to the accumulation of cellular damage over time (López‐Otín et al. 2013). Many hallmarks of aging impinge on cellular metabolism. For example, aging associated changes in signaling through insulin, IGF1, mTOR, and AMPK have been well established (López‐Otín et al. 2013), and these signaling pathways are regulators of multiple metabolic pathways, including glycolysis, amino acid metabolism, and ATP production (Hoxhaj and Manning 2020). Insulin resistance tends to increase with age, leading to alterations in whole‐body glucose homeostasis (Ehrhardt et al. 2019; Reynolds et al. 2019). Sirtuins, which influence longevity, also target many metabolic enzymes, including those in the mitochondrial electron transport chain, the TCA cycle, ketogenesis, and fatty acid oxidation (Houtkooper, Pirinen, and Auwerx 2012). Additionally, aging related decline in the function of mitochondria can also impact cellular metabolism, including impaired respiration, decreased ATP generation, and increased production of reactive oxygen species (Amorim et al. 2022; Green, Galluzzi, and Kroemer 2011; López‐Otín et al. 2013). These observations led us to hypothesize that glucose utilization by tissues may change during aging. Indeed, prior work has provided evidence for aging related changes in glucose metabolism. For example, FDG‐PET imaging of human brains suggests that glucose uptake and aerobic glycolysis are impaired during aging, particularly with neurodegenerative disease (Goyal et al. 2017). On the other hand, several mouse studies have suggested that aerobic glycolysis and lactate levels increase with age in some tissues, including the brain and skeletal muscle (Gomes et al. 2013; Ross et al. 2010). Profiling of aging animals has also revealed alterations in glucose metabolism gene expression and in levels of metabolites involved in central carbon and amino acid metabolism in multiple tissues (Houtkooper et al. 2011; Walters et al. 2018).

The intravenous infusion of stable isotope‐labeled nutrients can be a powerful tool for assessing nutrient utilization in tissues within live animals. This approach has helped expand our understanding of tumor metabolism, particularly by highlighting how in vivo tumor metabolism can be distinct from the metabolism of cultured cancer cells and how it varies across tissues (Altea‐Manzano et al. 2023; Davidson et al. 2016; Elia et al. 2019; Faubert et al. 2017; Hui et al. 2017; Lau et al. 2020; Yuneva et al. 2012). This method has also been used to explore normal physiology to understand the fuel preferences of different tissues (Hui et al. 2020, 2017; Jang et al. 2019, 2018). In this study, we used intravenous infusion of [U‐^13^C]‐glucose in young and old mice across three different mouse strains to examine how glucose utilization in normal tissues changes during aging. While minimal changes in glucose labeling were observed in tissues with age, several caveats are associated with the interpretation of these data. First, because changes to whole‐body metabolic physiology occur during aging, such as increased insulin resistance, this can lead to distinct responses by young versus old mice to the infusion of exogenous glucose. We optimized a [U‐^13^C]‐glucose infusion rate in young and old mice that minimizes elevations in blood glucose and insulin levels while still leading to reasonable tissue metabolite labeling (Figure 1), and we found that using different infusion rates can lead to distinct results in tissue metabolite labeling between young and old mice (Figure S2). However, despite efforts to determine an optimal infusion rate, we cannot rule out that young and old mice may still respond differently to the glucose infusions. Another caveat is that while steady‐state labeling of [U‐^13^C]‐glucose can provide data on relative differences in label incorporation into different metabolites, this approach does not directly measure differences in metabolic pathway fluxes. Therefore, our data argue against major shifts in fuel sources used by different tissues, but may not reflect more subtle changes in metabolic pathway fluxes. Finally, conclusions about tissue glucose utilization are complicated by inter‐ and intratissue metabolic interactions. For example, while labeling of glycolytic and TCA cycle intermediates from [U‐^13^C]‐glucose in the muscle can result from direct glucose uptake by muscle cells, indirect labeling can also occur through labeled intermediates that are produced by other organs and released into circulation. Indeed, it has been proposed that glucose may produce lactate that in turn fuels the TCA cycle in tissues (Hui et al. 2017). Similar interactions can occur between different cell types within the same tissue. Label incorporation can also be confounded by exchange flux, in which rapid substrate–product interconversion by reversible metabolic reactions can lead to labeling of downstream metabolites regardless of the net direction of those reactions (Buescher et al. 2015).

To our surprise, we found that glucose contribution to glycolysis, the TCA cycle, and amino acid metabolism in the tissues analyzed did not significantly change during aging in C57BL/6J, WSB/EiJ, and DO mice. The only observed difference in metabolite labeling was tissue specific, with brain tissues exhibiting distinct labeling patterns compared to plasma, liver, and gastrocnemius that are consistent with the brain being a more glucose‐avid tissue (Hui et al. 2017). Similarly, we found that levels of NAD^+^, NADH, and the NAD^+^/NADH ratio also did not change with healthy aging in our mouse cohorts. Given the limited sample size of aged mice analyzed, one possibility is that this study was not sufficiently powered to detect more subtle declines in NAD^+^ levels. Moreover, different approaches have been used to measure NAD^+^ and NADH levels in the literature, and differences in assay sensitivities could explain the lack of difference found in this study. Notably, however, a recent study also showed that the degree of aging associated NAD^+^ decline can vary across tissues, with minor decreases in NAD^+^, minimal changes in NADH, and minor or no changes in tissue NAD^+^/NADH ratios (McReynolds et al. 2021). Our conclusion that glucose carbon utilization, either directly or indirectly, as a fuel source for central carbon metabolism is robust in healthy aging mice may be unexpected, particularly in light of well‐documented evidence for decline in mitochondrial function with age. One possibility is that the contribution of glucose to central carbon metabolism relative to other potential nutrients, such as amino acids and lipids, does not change with age, even if metabolic flux involving mitochondrial metabolism declines. Additionally, we intentionally selected older animals that were overtly healthy for analysis, and it is possible that this may have also selected for older mice with healthier mitochondria. Nevertheless, these data still argue that chronologically older mice do not exhibit major shifts in glucose utilization for central carbon and amino acid metabolism.

Despite a lack of age‐associated changes observed in central carbon metabolism, our data suggest that lipid metabolism may be altered with aging, particularly in brain tissue. Interestingly, many of the lipid metabolism reactions associated with the alterations regenerate NAD^+^ from NADH. In all three mouse strains, fatty acid desaturation, which converts NADH to NAD^+^ in the process of synthesizing unsaturated fatty acids, appears to be increased in older tissues, particularly the brain. These observations corroborate previous studies that found membrane fatty acid desaturation increases with age in many tissues. Highly unsaturated fatty acids are more susceptible to lipid peroxidation by reactive oxygen species, which is hypothesized to contribute to aging related cellular damage (Naudí et al. 2013). Similarly, we also found in all three mouse strains that aging brain tissues had elevated levels of sphingolipids, with additional evidence suggesting aging associated increases in the activities of DEGS1 and FA2H, which are coupled to the oxidation of NAD(P)H to NAD(P)^+^. These observations are also consistent with prior studies observing a similar increase in the levels of sphingolipids in aging brain tissues, particularly species containing unsaturated and 2‐hydroxy fatty acids (Couttas et al. 2018; Kishimoto and Radin 1959). Ceramides are lipids that can be proapoptotic and have been implicated in aging related pathologies such as neurodegeneration and insulin resistance (Chaurasia and Summers 2015).

Why aging tissues might upregulate fatty acid desaturation and sphingolipid metabolism, particularly when these processes generate potentially pathological lipid species, remains unclear. One possibility is that if subtle declines in NAD^+^ levels are occurring during aging, this may be associated with reductive stress (i.e., an accumulation of NADH) that upregulates NAD^+^‐producing reactions. Alternatively, we speculate that these NAD^+^‐producing processes could be a mechanism for tissues to maintain NAD^+^/NADH homeostasis. In normal tissues in young animals, fully functional mitochondrial respiration may enable cells to maintain a NAD^+^/NADH ratio that supports tissue function. As aging leads to progressive mitochondrial dysfunction, cells may need to shift to alternative metabolic reactions that regenerate NAD^+^ to maintain their NAD^+^/NADH ratio, including lipid metabolism reactions such as fatty acid desaturation and sphingolipid synthesis. These reactions could therefore serve as a compensatory system that buffers against mitochondria‐associated declines in NAD^+^ production. Whether the availability of fatty acid and lipid precursors for desaturation and sphingolipid synthesis is high enough to serve as a significant source for NAD^+^ regeneration is difficult to quantify, but evidence for ongoing membrane remodeling and lipid turnover in tissues (Wang and Tontonoz 2019) make it plausible that sustained activity of these lipid metabolism reactions is possible. Moreover, it has been reported that polyunsaturated fatty acid synthesis through the fatty acid desaturases FADS1 and FADS2 are upregulated to generate NAD^+^ in response to inhibition of mitochondrial respiration (Kim et al. 2019). Nevertheless, our data does not provide any direct mechanistic evidence for such a NAD^+^/NADH buffering system. One potential prediction of this model is that older animals will have already engaged alternative NAD^+^‐generating reactions to maintain the NAD^+^/NADH ratio, and compared to younger animals, older animals will be more sensitive to further stresses such as mitochondrial electron transport chain inhibitors that perturb this ratio. Interestingly, the mitochondrial complex I inhibitor rotenone has been used in rat and mouse models to induce features of Parkinson's disease (Betarbet et al. 2000), and administration of rotenone to aged mice leads to more severe Parkinson's‐like symptoms compared to young mice (Weetman et al. 2013). As further work is done to characterize how various metabolic pathways change during aging, it will be interesting to focus on reactions that are capable of regenerating NAD^+^ to understand if they might be regulated to help maintain NAD^+^/NADH homeostasis.

Finally, we note that the results presented here are specific to the three mouse strains analyzed in this study, which have known distinct metabolic features. For example, WSB/EiJ mice are resistant to high‐fat diet‐induced obesity and have a unique insulin secretion and sensitivity phenotype (Lee et al. 2011). DO mice are generated by breeding multiple inbred strains together, with each inbred strain having unique characteristics such as increased diabetes risk, increased cancer risk, and increased susceptibility to obesity (Bogue, Churchill, and Chesler 2015). We aimed to identify common metabolic features associated with aging across these mouse strains, and indeed we observed a consistent lack of change to glucose contribution to central carbon and amino acid metabolism, as well as increased fatty acid desaturation and sphingolipid metabolism in the brain. We observed some strain‐specific metabolite features; for example, there were strain‐specific differences in how the total levels of TCA cycle intermediates and amino acids changed with age, arguing these changes are not general features of murine aging. Given that both genetic background and sex among different mouse strains influence tissue metabolic activity (Bachmann et al. 2022), it is possible that applying our analyses to additional mouse strains might reveal additional aging associated or strain‐specific metabolic changes. Finally, whether the lack of changes observed in glucose metabolism, or the alterations to lipid metabolism, observed in this study apply to human aging remains an open question. Nevertheless, the data presented here provides a resource that characterizes metabolism with age across three diverse strains of mice.

## 4 Materials and Methods

### 4.1 Animal Studies

All experiments conducted in this study were approved by the MIT Committee on Animal Care (IACUC). Male C57BL/6J mice were obtained from The Jackson Laboratory (000664) or Calico Labs and aged in house. Male 3‐month‐old and 2‐year‐old WSB/EiJ and diversity outbred (DO) mice were obtained from Calico Labs. All animals were housed at ambient temperature and humidity (18°C–23°C, 40%–60% humidity) with a 12‐h light and 12‐h dark cycle and cohoused with ad libitum access to water. Data were collected from distinct animals, where *n* represents biologically independent samples. Only mice with a body score condition of ≥ 2 with no overt signs of disease were used for experiments. Statistical methods were not performed to predetermine sample size.

### 4.2 Glucose Infusion

Infusion of [U‐^13^C]‐glucose (Cambridge Isotope Laboratories) was performed as previously described (Davidson et al. 2016; Lau et al. 2020). A catheter was surgically implanted into the jugular vein of animals 3–4 days prior to infusion. Mice were fasted for 4 h prior to starting infusions, which were conducted in conscious, free‐moving animals at the indicated infusion rates for up to 6 h.

### 4.3 Blood Glucose and Plasma Insulin Measurements

Blood glucose levels were measured using a Contour glucose meter (Ascensia Diabetes Care). Plasma insulin was measured with an ultrasensitive mouse insulin ELISA (Crystal Chem #90080).

### 4.4 Tissue and Plasma Polar Metabolite Extraction

Snap‐frozen tissues were ground into powder, and polar metabolites were extracted with a 5:3:5 ratio of ice‐cold HPLC‐grade methanol:water:chloroform containing norvaline as an internal standard. For plasma, blood collected from animals was immediately placed in EDTA tubes (Sarstedt 41.1395.105) and centrifuged to separate plasma. Plasma (10 μL) was extracted with 300 μL of ice‐cold methanol. Samples were vortexed for 15 min at 4°C and centrifuged at maximum speed for 10 min at 4°C. The aqueous polar metabolite fraction was dried under nitrogen gas and frozen at −80°C until analysis.

### 4.5 Tissue and Plasma Lipid Extraction for Fatty Acid Analysis

Snap‐frozen tissues were ground into powder using a mortar and pestle. Tissue powder was then weighed into glass vials (Thermo Fisher C4010‐1, C4010‐60BLK). Blood collected from animals was immediately placed in EDTA tubes (Sarstedt 41.1395.105) and centrifuged to separate plasma. Lipids were extracted in 1.5 mL dichloromethane:methanol (containing 25 mg/L butylated hydroxytoluene, Millipore Sigma B1378): 0.88% KCl (w/v) (8:4:3), vortexed for 15 min at 4°C, and centrifuged at maximum speed for 10 min at 4°C. The extraction buffer contained either 0.7 μg/mL tridecanoic acid or 0.7 μg/mL *cis*‐10‐heptadecenoic acid as internal standards. Lipids (organic fraction) were transferred to glass vials, dried under nitrogen gas, and immediately processed for analysis.

### 4.6 Gas Chromatography–Mass Spectrometry (GC–MS) Analysis of Polar Metabolites

Polar metabolites were analyzed by GC–MS as described previously (Lien et al. 2021). Dried and frozen metabolite extracts were derivatized with 16 μL of MOX reagent (Thermo Fisher TS‐45950) for 60 min at 37°C, followed by derivatization with 20 μL of N‐*tert*‐butyldimethylsilyl‐N‐methyltrifluoroacetamide with 1% *tert*‐butyldimethylchlorosilane (Millipore Sigma 375934) for 30 min at 60°C. Derivatized samples were analyzed by GC–MS, using a DB‐35MS column (Agilent Technologies 122‐3832) installed in an Agilent 7890B gas chromatograph coupled to an Agilent 5997B mass spectrometer. Helium was used as the carrier gas at a constant flow rate of 1.2 mL/min. One microliter of sample was injected in split mode (1:10) at 270°C. After injection, the GC oven was held at 100°C for 1 min, increased to 105°C at 2.5°C/min, held at 105°C for 2 min, increased to 250°C at 3.5°C/min, and then ramped to 320°C at 20°C/min. The MS system operated under electron impact ionization at 70 eV, and the MS source and quadrupole were held at 230°C and 150°C, respectively. The detector was used in scanning mode with an ion range of 100–650 *m/z*.

Glucose was analyzed by GC–MS as described previously (Antoniewicz, Kelleher, and Stephanopoulos 2011). Dried and frozen metabolite extracts were derivatized with 50 μL of 2% (w/v) hydroxylamine hydrochloride in pyridine (Millipore Sigma) for 60 min at 90°C, followed by derivatization with 100 μL of propionic anhydride (Millipore Sigma) for 30 min at 60°C. Derivatized samples were then dried under nitrogen gas and resuspended in 100 μL of ethyl acetate (Millipore Sigma) in glass GC–MS vials. Samples were analyzed by GC–MS as described earlier, except helium was used as the carrier gas at a constant flow rate of 1.1 mL/min, and 1 μL of sample was injected in splitless mode at 250°C. After injection, the GC oven was held at 80°C for 1 min, ramped to 280°C at 20°C/min, and held at 280°C for 4 min.

Total ion counts were determined by integrating appropriate ion fragments for each metabolite using El‐Maven software (Elucidata). Mass isotopolog distributions were corrected for natural abundance using IsoCorrectoR (Heinrich et al. 2018). Metabolite data were normalized to the internal standard and biofluid volumes/tissue weights.

### 4.7 Gas Chromatography–Mass Spectrometry (GC–MS) Analysis of Fatty Acid Methyl Esters

Fatty acid methyl esters (FAMEs) were analyzed by GC–MS as described previously (Lien et al. 2021). Dried lipid extracts were resuspended in 100 μL of toluene in glass vials and derivatized with 200 μL of 2% sulfuric acid in methanol overnight at 50°C. After derivatization, 500 μL of 5% NaCl was added, and FAMEs were extracted twice with 500 μL of hexane. Samples from animal tissues or biofluids were cleaned up with Bond Elut LRC‐Florisil columns (Agilent Technologies 12,113,049). Columns were preconditioned with 3 mL of hexane, and then the FAME extracts in hexane were added to the column. FAMEs were finally eluted twice with 1 mL of hexane:diethyl ether (95:5 v/v), dried under nitrogen gas, and resuspended in hexane for GC–MS analysis. GC–MS was conducted with a DB‐FastFAME column (Agilent Technologies G3903‐63011) installed in an Agilent 7890A gas chromatograph coupled to an Agilent 5975C mass spectrometer. Helium was used as the carrier gas at a constant pressure of 14 psi. One microliter of sample was injected in splitless mode at 250°C. After injection, the GC oven was held at 50°C for 0.5 min, increased to 194°C at 25°C/min, held at 194°C for 1 min, increased to 245°C at 5°C/min, and held at 245°C for 3 min. The MS system operated under electron impact ionization at 70 eV, and the MS source and quadrupole were held at 230°C and 150°C, respectively. The detector was used in scanning mode with an ion range of 104–412 *m/z*. Total ion counts were determined by integrating appropriate ion fragments for each FAME using El‐Maven software (Elucidata). Metabolite data were background corrected using a blank sample and normalized to the internal standard and biofluid volumes/tissue weights.

### 4.8 NAD ^+^/NADH Measurements

Snap‐frozen tissues were ground into powder using a mortar and pestle. Tissue powder was weighed and extracted in 200 μL of ice‐cold lysis buffer (1% dodecyltrimethylammonium bromide in 0.2 N of NaOH diluted 1:1 with PBS) per 10 mg tissue, snap frozen in liquid nitrogen, and frozen at −80°C. NAD^+^ and NADH were measured using a protocol adapted from the manual of the NAD^+^/NADH‐Glo Assay Kit (Promega, G9072), as previously described (Li et al. 2022). Briefly, to measure NAD^+^, 20 μL of lysate was diluted with 20 μL of lysis buffer and 20 μL 0.4 N HCl, and incubated at 60°C for 15 min. These acidic conditions selectively degrade NADH. To measure NADH, 20 μL of lysate was incubated at 75°C for 30 min, under basic conditions that selectively degrade NAD^+^. After incubation, samples were quenched with 20 μL of neutralizing solution (0.5 M Tris base for NAD^+^ samples and 0.25 M Tris in 0.2 N HCl for NADH samples). The protocol in the manual of the NAD^+^/NADH‐Glo Assay Kit was then followed to individually measure NAD^+^ and NADH levels using a luminometer (Tecan Infinite M200Pro). External NAD^+^ and NADH standards were not used in each assay run; rather, prior to each analysis, NAD^+^ and NADH were measured in one tissue sample at various dilutions to determine the appropriate tissue lysate dilution that resulted in luminescence signal within the linear range of detection for both NAD^+^ and NADH. Therefore, NAD^+^ and NADH levels were measured as arbitrary luminescence units that are normalized to tissue weight, and displayed in the figures as fold changes relative to the value detected in 3‐month‐old mice. To calculate the absolute NAD^+^/NADH ratio, the NAD^+^ luminescence value was divided by the NADH luminescence value, and this ratio was multiplied by 2 (because NADH samples were diluted 1:2 and NAD^+^ samples were diluted 1:4 in the assay protocol).

### 4.9 Extraction of Tissue and Plasma for LC–MS Metabolomics and Lipidomics

Per sample, 50 μL of plasma was used, and 35 mg of homogenized tissue was used for extraction. Sample were placed in 2 mL glass vial and extracted for LC–MS‐based lipidomics and metabolomics analysis using methyl‐*tert*‐butyl ether liquid–liquid extraction (MTBE‐LLE) (Matyash et al. 2008). In short, 350 μL of water and 400 μL of methanol containing internal standards (100 μL of LipidSplash standards [Avanti Polar Lipids], 0.5 μg/mL Tyr‐d5, 0.5 μg/mL Arg‐^15^N_4_, and 125 ng/mL Benzonic acid‐d5) was added to the sample, vortexed for 30 s, and incubated on ice for 15 min. To the mixture, 800 μL of MTBE was added, vortexed for 30 s, incubated on ice for another 15 min, and centrifuged at 3500 rpm for 10 min at 4°C. Lipids partitioned in the top layer was collected into a separate vial, and the extraction process was repeated again with an additional 600 μL of MTBE. After incubating and centrifuging, the second organic layer was collected and combined in the first lipid vial. The remaining aqueous layer containing polar metabolites was transferred to a glass vial. Both extractions were dried under nitrogen at 4°C. For lipidomics analysis, samples were resuspended in 200 μL of butanol/methanol/water (2:1:1, v/v/v), and polar metabolites were resuspended in 80% acetonitrile for LC–MS analysis.

### 4.10 LC–MS Analysis of Polar Metabolites

Metabolomics samples were analyzed in both positive and negative ESI–LC–MS methods on Vanquish UPLCs coupled to Q‐Exactive Plus mass spectrometers. In positive mode, metabolites were separated using a SeQuant ZIC‐pHILIC column (5 μm, 200 Å, 150 × 2.1 mm). Mobile phase A was 20 mM ammonium carbonate in water (pH 9.2) and mobile phase B was acetonitrile at a flow rate of 150 μL/min and the gradient was *t* = −6, 80% B; *t* = 0, 80% B; *t* = 2.5, 73% B; *t* = 5, 65% B, *t* = 7.5, 57% B; *t* = 10, 50% B; *t* = 15, 35% B; *t* = 20; 20% B; *t* = 22, 15% B; *t* = 22.5, 80% B; *t* = 24; 80% B. Data were acquired using data‐dependent acquisition (DDA) mode with the following parameters: resolution = 70,000, AGC target = 3.00 × 10^6^, maximum IT (ms) = 100, scan range = 70–1050. The MS2 parameters were as follows: resolution = 17,500, AGC target = 1.00 × 10^5^, maximum IT (ms) = 50, loop count = 6, isolation window (*m*/*z*) = 1, (N)CE = 20, 40, 80; underfill ratio = 1.00%, Apex trigger(s) = 3–10, dynamic exclusion(s) = 25.

In negative mode, metabolites were separated using a reverse phase ion‐pairing chromatographic method using an Agilent Extend C18 RRHD column (1.8 μm, 80 Å, 2.1 × 150 mm). Mobile phase A was 10 mM tributylamine, 15 mM acetic acid in 97:3 water:methanol, pH 4.95; mobile phase B was methanol at a flow rate of 200 μL/min. The gradient was *t* = −4, 0% B; *t* = 0, 0% B; *t* = 5; 20% B; *t* = 7.5, 20% B; *t* = 13, 55% B; *t* = 15, 95% B; *t* = 18.5, 95% B; *t* = 19, 0% B; *t* = 22, 0% B. Data were acquired in data‐dependent acquisition (DDA) mode with the following parameters: resolution = 70,000, AGC target = 1.00 × 10^6^, maximum IT (ms) = 100, scan range = 70–1050. The MS/MS parameters were as follows: resolution = 17,500, AGC target = 1.00 × 10^5^, maximum IT (ms) = 50, loop count = 6, isolation window (*m*/*z*) = 1, (N)CE = 20, 50, 100; underfill ratio = 1.00%, Apex trigger(s) = 3–12, dynamic exclusion(s) = 20.

### 4.11 LC–MS Analysis of Lipids

Lipidomics samples were analyzed in both positive and negative ion mode using the same LC–MS method consisting of a Vanquish UPLC coupled to a Q‐Exactive Plus mass spectrometer. Lipids were separated using a Thermo Fisher Scientific Accucore C30 column (2.6 μm, 150 Å, 2.1 × 250 mm) at a flow rate of 200 μL/min. For the LC method, mobile phase A was 20 mM ammonium formate in 60:40 acetonitrile:water + 0.25 μM medronic acid, and mobile phase B was 20 mM ammonium formate in 90:10 isopropanol:acetonitrile + 0.25 μM medronic acid. The gradient was *t* = −7, 30% B, *t* = 0, 30% B, *t* = 7, 43% B, *t* = 12, 65% B, *t* = 30, 70% B, *t* = 31, 88% B, *t* = 51, 95% B, *t* = 53, 100% B, *t* = 55, 100% B, *t* = 55.1, 30% B, *t* = 60, 30% B. The mass spectrometer settings were as follows: data‐dependent acquisition (DDA) was performed with the following parameters: resolution = 140,000, AGC target = 3.00 × 10^6^, maximum IT (ms) = 100, scan range = 200–2000. The MS2 parameters were as follows: resolution = 17,500, AGC target = 3.00 × 10^6^, maximum IT (ms) = 150, loop count = 8, isolation window (*m*/*z*) = 1, (N)CE = 20, 30, 40; underfill ratio = 1.00%, Apex trigger(s) = 5–30, dynamic exclusion(s) = 15 s.

### 4.12 LC–MS Data Analysis

RAW files were converted to mzML files using msconvert from ProteoWizard, using vendor centroiding on all scans, and analyzed using MAVEN2 software (Seitzer, Bennett, and Melamud 2022) against *in‐house* libraries.

### 4.13 Statistics and Reproducibility

Sample sizes, reproducibility, and statistical tests used for each figure are denoted in the figure legends. All graphs were generated using GraphPad Prism 9.

## Author Contributions

Evan C. Lien, Laura V. Danai, Allison N. Lau, Bryson D. Bennett, and Matthew G. Vander Heiden conceived the project. Evan C. Lien, Ngoc Vu, Anna M. Westermark, Laura V. Danai, Allison N. Lau, Yetiş Gültekin, and Matthew A. Kukurugya performed the experiments and analyzed data. Evan C. Lien and Matthew G. Vander Heiden wrote the manuscript with input from all authors.

## Conflicts of Interest

M.G.V.H. is a scientific advisor for Agios Pharmaceuticals, iTeos Therapeutics, Sage Therapeutics, Lime Therapeutics, Pretzel Therapeutics, Droia Ventures, MPM Captital, and Auron Therapeutics. A.N.L. is a current employee of Pfizer Inc., and A.M.W. is a current employee of a Flagship Ventures start‐up company; however, all work was performed while at MIT.

## Supporting information


**Figure S1.** A 6‐h infusion is required to reach steady‐state labeling in C57BL/6J mouse tissues. C57BL/6J mice were infused with [U‐^13^C]‐glucose at 0.4 mg/min for 0.5 h (*n* = 2), 2 h (*n* = 3), 4 h (*n* = 3), and 6 h (*n* = 2 for plasma, *n* = 3 for tissues). (A) Plasma glucose enrichment. [M + 3] fractional labeling of pyruvate, lactate, and alanine (B), [M + 2] fractional labeling of the indicated TCA cycle metabolites (C), and fractional labeling of the indicated amino acids (D) in plasma, liver, gastrocnemius muscle, and brain tissues over time. Data are presented as mean ± SEM.

**Figure S2.** [U‐^13^C]‐Glucose infusion rate differences can impact tissue metabolite labeling patterns in young versus old C57BL/6J mice. C57BL/6J mice, 3‐month‐old (*n* = 3) versus 24‐month‐old (*n* = 6), were infused with [U‐^13^C]‐glucose at 30 mg/kg/min for 4 h. [M + 3] fractional labeling of pyruvate (A) and lactate (B) in the indicated tissues. Data are presented as mean ± SEM. Comparisons were made using a two‐tailed Student’s *t* test. *p< 0.05, **p< 0.01, ***p< 0.001.

**Figure S3.** Glucose contribution to glycolysis is robust in aging WSB/EiJ and DO mice. WSB/EiJ mice, 3‐month‐old (*n* = 6) versus 24‐month‐old (*n* = 5), were infused with [U‐^13^C]‐glucose at 0.4 mg/min for 6 h. DO mice, 3‐month‐old (*n* = 6), 24‐month‐old (*n* = 4), and 30‐month‐old (*n* = 4), were infused with [U‐^13^C]‐glucose at 0.4 mg/min for 6 h. [M + 3] fractional labeling of pyruvate (A), lactate (B), and alanine (C) in the indicated tissues from WSB/EiJ mice. Relative levels of pyruvate (D), lactate (E), and alanine (F) in the indicated tissues from WSB/EiJ mice. [M + 3] fractional labeling of pyruvate (G), lactate (H), and alanine (I) in the indicated tissues from DO mice. Relative levels of pyruvate (J), lactate (K), and alanine (L) in the indicated tissues from DO mice. Data are presented as mean ± SEM. Relative metabolite levels (D–F, J–L) represent mass spectrometry peak areas that are normalized to an internal standard and tissue weight, before being normalized relative to the average value in 3‐month‐old mice. Comparisons were made using a two‐tailed Student’s *t* test. *p< 0.05, **p< 0.01.

**Figure S4.** Glucose contribution to the TCA cycle is robust in aging C57BL/6J mice. C57BL/6J mice, 3‐month‐old (*n* = 4) versus 24‐month‐old (*n* = 4), were infused with [U‐^13^C]‐glucose at 0.4 mg/min for 6 h. Mass isotopomer distributions of α‐ketoglutarate (αKG), succinate, fumarate, and aspartate in plasma (A), liver (B), gastrocnemius muscle (C), and brain (D) tissues. Data are presented as mean ± SEM.

**Figure S5.** Glucose contribution to the TCA cycle is robust in aging WSB/EiJ mice. WSB/EiJ mice, 3‐month‐old (*n* = 6) versus 24‐month‐old (*n* = 5), were infused with [U‐^13^C]‐glucose at 0.4 mg/min for 6 h. Relative levels of TCA cycle metabolites in plasma (A), liver (B), gastrocnemius muscle (C), and brain (D) tissues. Relative metabolite levels represent mass spectrometry peak areas that are normalized to an internal standard and tissue weight, before being normalized relative to the average value in 3‐month‐old mice. Mass isotopomer distributions of the indicated TCA cycle metabolites in plasma (E), liver (F), gastrocnemius muscle (G), and brain (H) tissues. Data are presented as mean ± SEM. Comparisons were made using a two‐tailed Student’s *t* test. *p< 0.05, **p< 0.01.

**Figure S6.** Glucose contribution to the TCA cycle is robust in aging DO mice. DO mice, 3‐month‐old (*n* = 6), 24‐month‐old (*n* = 4), and 30‐month‐old (*n* = 4), were infused with [U‐^13^C]‐glucose at 0.4 mg/min for 6 h. Relative levels of TCA cycle metabolites in plasma (A), liver (B), gastrocnemius muscle (C), and brain (D) tissues. Relative metabolite levels represent mass spectrometry peak areas that are normalized to an internal standard and tissue weight, before being normalized relative to the average value in 3‐month‐old mice. Mass isotopomer distributions of the indicated TCA cycle metabolites in plasma (E), liver (F), gastrocnemius muscle (G), and brain (H) tissues. Data are presented as mean ± SEM. Comparisons were made using a two‐tailed Student’s *t* test. *p< 0.05.

**Figure S7.** Glucose contribution to amino acid metabolism is robust in aging WSB/EiJ mice. WSB/EiJ mice, 3‐month‐old (*n* = 6) versus 24‐month‐old (*n* = 5), were infused with [U‐^13^C]‐glucose at 0.4 mg/min for 6 h. (A) Relative levels of the indicated amino acids in plasma, liver, gastrocnemius muscle, and brain tissues. Relative metabolite levels represent mass spectrometry peak areas that are normalized to an internal standard and tissue weight, before being normalized relative to the average value in 3‐month‐old mice. Fractional labeling of [M + 2] and [M + 3] asparagine (B), [M + 2] and [M + 3] glutamine (C), [M + 2] and [M + 3] glutamate (D), [M + 2] and [M + 3] proline (E), [M + 3] and [M + 1] serine (F), and [M + 2] glycine (G) in the indicated tissues. Data are presented as mean ± SEM. Comparisons were made using a two‐tailed Student’s *t* test. *p< 0.05, **p< 0.01, ***p< 0.001, ****p< 0.0001.

**Figure S8.** Glucose contribution to amino acid metabolism is robust in aging DO mice. DO mice, 3‐month‐old (*n* = 6), 24‐month‐old (*n* = 4), and 30‐month‐old (*n* = 4), were infused with [U‐^13^C]‐glucose at 0.4 mg/min for 6 h. (A) Relative levels of the indicated amino acids in plasma, liver, gastrocnemius muscle, and brain tissues. Relative metabolite levels represent mass spectrometry peak areas that are normalized to an internal standard and tissue weight, before being normalized relative to the average value in 3‐month‐old mice. Fractional labeling of [M + 2] and [M + 3] asparagine (B), [M + 2] and [M + 3] glutamine (C), [M + 2] and [M + 3] glutamate (D), [M + 2] and [M + 3] proline (E), [M + 3] and [M + 1] serine (F), and [M + 2] glycine (G) in the indicated tissues. Data are presented as mean ± SEM. Comparisons were made using a two‐tailed Student’s *t* test. *p< 0.05.

**Figure S9.** Polar metabolite profiling does not reveal strong age‐dependent changes in metabolite levels. Polar metabolite levels were measured by LC–MS in tissues from uninfused 3‐month‐old (*n* = 9), 12‐month‐old (*n* = 3), and 24‐month‐old (*n* = 3) C57BL/6J mice. Data were analyzed using MetaboAnalyst. Heat maps of metabolite levels from liver (A), gastrocnemius muscle (B), and brain (C) tissues. Principal component analysis of metabolite levels from liver (D), gastrocnemius muscle (E), and brain (F) tissues.

**Figure S10.** Fatty acid desaturation increases in tissues from aging WSB/EiJ mice. Relative levels of the indicated fatty acids in plasma (A), liver (B), gastrocnemius muscle (C), and brain (D) tissues from young versus old WSB/EiJ mice. Relative fatty acid levels represent mass spectrometry peak areas that are normalized to an internal standard and tissue weight, before being normalized relative to the average value in 3‐month‐old mice. Relative fatty acid desaturation ratios in plasma (E), liver (F), gastrocnemius muscle (G), and brain (H) tissues from young versus old WSB/EiJ mice. Relative fatty acid ratios are shown normalized to the average value in 3‐month‐old mice. 3 months *n* = 6, 24 months *n* = 5. Data are presented as mean ± SEM. Comparisons were made using a two‐tailed Student’s *t* test. *p< 0.05, **p< 0.01, ***p< 0.001, ****p< 0.0001.

**Figure S11.** Fatty acid desaturation increases in tissues from aging DO mice. Relative levels of the indicated fatty acids in plasma (A), liver (B), gastrocnemius muscle (C), and brain (D) tissues from young versus old DO mice. Relative fatty acid levels represent mass spectrometry peak areas that are normalized to an internal standard and tissue weight, before being normalized relative to the average value in 3‐month‐old mice. Relative fatty acid desaturation ratios in plasma (E), liver (F), gastrocnemius muscle (G), and brain (H) tissues from young versus old DO mice. Relative fatty acid ratios are shown normalized to the average value in 3‐month‐old mice. 3 months *n* = 6, 24 months *n* = 4, 30 months *n* = 4. Data are presented as mean ± SEM. Comparisons were made using a two‐tailed Student’s *t* test. *p< 0.05, **p< 0.01, ***p< 0.001, ****p< 0.0001.

**Figure S12.** Aging brain tissue exhibits changes in levels of d18:0‐, d18:1‐, and d18:2‐containing sphingolipid species. Heat maps of relative levels of d18:0‐, d18:1‐, and d18:2‐containing sphingolipid species in brain tissues from young versus old C57BL/6J (A), WSB/EiJ (B), and DO (C) mice. Heat map scale bars represent *z* scores. C57BL/6J: 3 months *n* = 3, 24 months *n* = 3. WSB/EiJ: 3 months *n* = 6, 24 months *n* = 5. DO: 3 months *n* = 6, 24 months *n* = 4, 30 months *n* = 4.

**Figure S13.** Aging brain tissue exhibits changes in levels of sphingolipid species that contain 2‐hydroxylated fatty acids. Heat maps of relative levels of sphingolipid species that contain 2‐hydroxylated fatty acids in brain tissues from young versus old C57BL/6J (A), WSB/EiJ (B), and DO (C) mice. Heat map scale bars represent *z* scores. C57BL/6J: 3 months *n* = 3, 24 months *n* = 3. WSB/EiJ: 3 months *n* = 6, 24 months *n* = 5. DO: 3 months *n* = 6, 24 months *n* = 4, 30 months *n* = 4.

**Table S1.** Infusion rate parameters for optimizing [U‐^13^C]‐glucose infusions.

**Table S2.** Body weights (g) of all infused mice.

**Table S3.** Metabolite labeling data normalized to plasma glucose enrichment for C57BL/6J mice.

**Table S4.** Metabolite labeling data normalized to plasma glucose enrichment for WSB/EiJ mice.

**Table S5.** Metabolite labeling data normalized to plasma glucose enrichment for DO mice.

## Data Availability

All data are included in the manuscript and are available upon reasonable request from the corresponding authors.
