# Supplementary figures and images for: Effects of Aging on Glucose and Lipid Metabolism in Mice
Source: Aging Cell. 2024 Dec 27;24(4):e14462. doi: 10.1111/acel.14462 (PMC11984682; doi:10.1111/acel.14462)

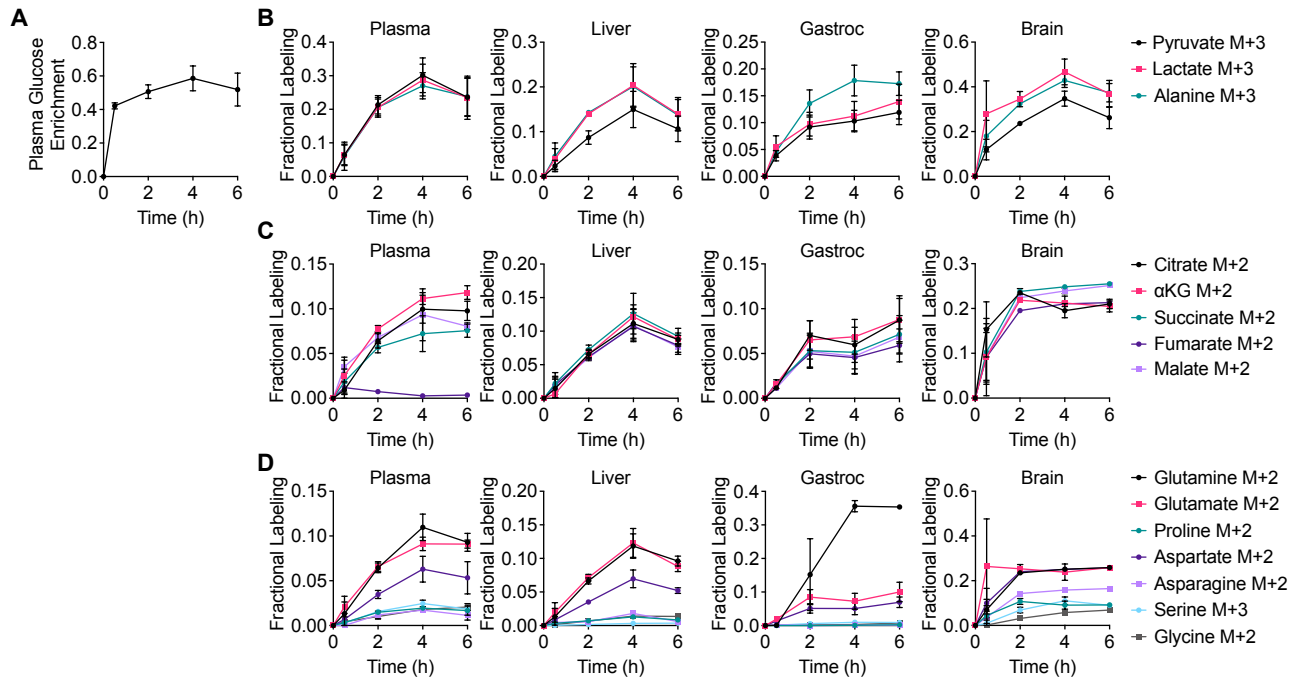

**Figure S1**

Supplement: Supplementary file 1 — Figure S1. A 6‐h infusion is required to reach steady‐state labeling in C57BL/6J mouse tissues. C57BL/6J mice were infused with [U‐13C]‐glucose at 0.4 mg/min for 0.5 h (n = 2), 2 h (n = 3), 4 h (n = 3), and 6 h (n = 2 for plasma, n = 3 for tissues). (A) Plasma glucose enrichment. [M + 3] fractional labeling of pyruvate, lactate, and alanine (B), [M + 2] fractional labeling of the indicated TCA cycle metabolites (C), and fractional labeling of the indicated amino acids (D) in plasma, liver, gastrocnemius muscle, and brain tissues over time. Data are presented as mean ± SEM. [file ACEL-24-e14462-s004.pdf]

**A**

Pyruvate M+3

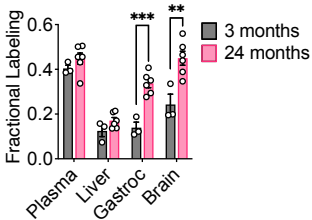**B**

Lactate M+3

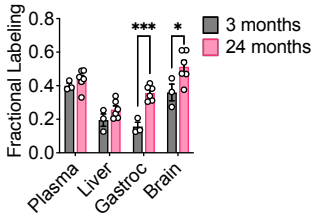**Figure S2**

Supplement: Supplementary file 2 — Figure S2. [U‐13C]‐Glucose infusion rate differences can impact tissue metabolite labeling patterns in young versus old C57BL/6J mice. C57BL/6J mice, 3‐month‐old (n = 3) versus 24‐month‐old (n = 6), were infused with [U‐13C]‐glucose at 30 mg/kg/min for 4 h. [M + 3] fractional labeling of pyruvate (A) and lactate (B) in the indicated tissues. Data are presented as mean ± SEM. Comparisons were made using a two‐tailed Student’s t test. *p< 0.05, **p< 0.01, ***p< 0.001. [file ACEL-24-e14462-s010.pdf]

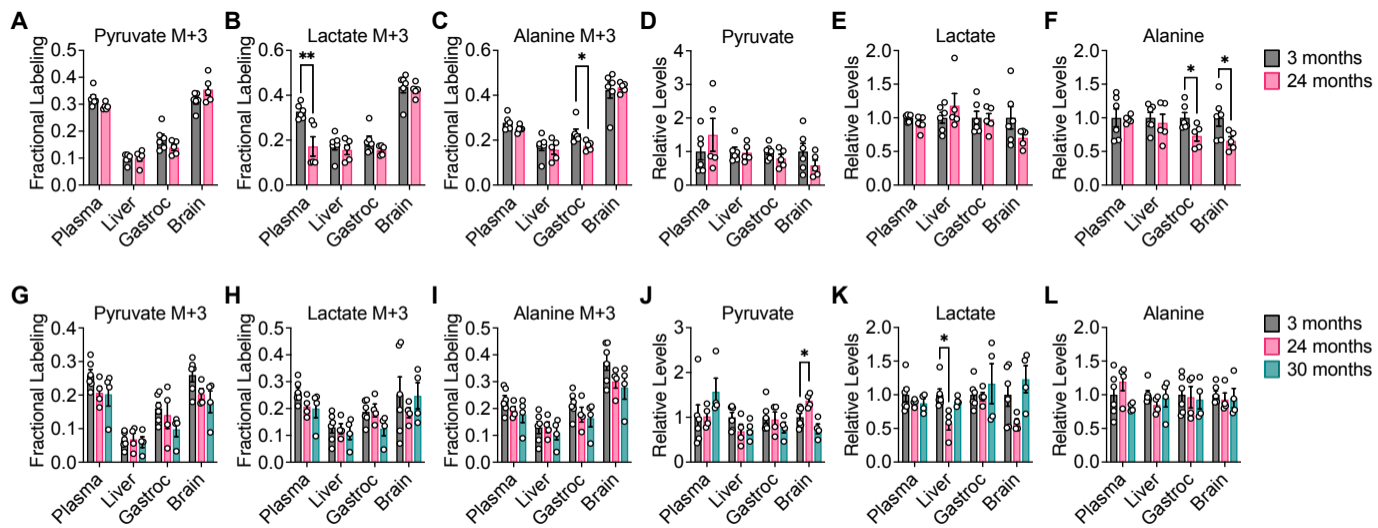

**Figure S3**

Supplement: Supplementary file 3 — Figure S3. Glucose contribution to glycolysis is robust in aging WSB/EiJ and DO mice. WSB/EiJ mice, 3‐month‐old (n = 6) versus 24‐month‐old (n = 5), were infused with [U‐13C]‐glucose at 0.4 mg/min for 6 h. DO mice, 3‐month‐old (n = 6), 24‐month‐old (n = 4), and 30‐month‐old (n = 4), were infused with [U‐13C]‐glucose at 0.4 mg/min for 6 h. [M + 3] fractional labeling of pyruvate (A), lactate (B), and alanine (C) in the indicated tissues from WSB/EiJ mice. Relative levels of pyruvate (D), lactate (E), and alanine (F) in the indicated tissues from WSB/EiJ mice. [M + 3] fractional labeling of pyruvate (G), lactate (H), and alanine (I) in the indicated tissues from DO mice. Relative levels of pyruvate (J), lactate (K), and alanine (L) in the indicated tissues from DO mice. Data are presented as mean ± SEM. Relative metabolite levels (D–F, J–L) represent mass spectrometry peak areas that are normalized to an internal standard and tissue weight, before being normalized relative to the average value in 3‐month‐old mice. Comparisons were made using a two‐tailed Student’s t test. *p< 0.05, **p< 0.01. [file ACEL-24-e14462-s013.pdf]

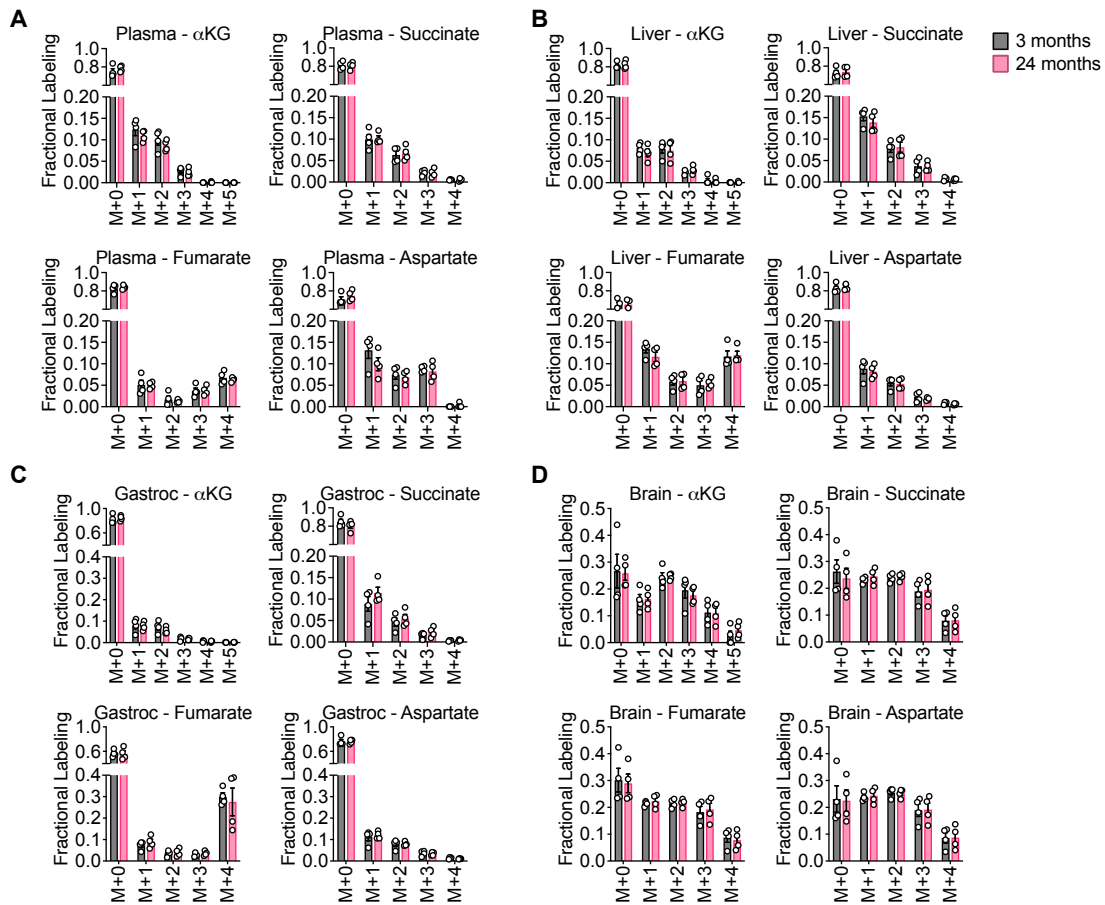

**Figure S4**

Supplement: Supplementary file 4 — Figure S4. Glucose contribution to the TCA cycle is robust in aging C57BL/6J mice. C57BL/6J mice, 3‐month‐old (n = 4) versus 24‐month‐old (n = 4), were infused with [U‐13C]‐glucose at 0.4 mg/min for 6 h. Mass isotopomer distributions of α‐ketoglutarate (αKG), succinate, fumarate, and aspartate in plasma (A), liver (B), gastrocnemius muscle (C), and brain (D) tissues. Data are presented as mean ± SEM. [file ACEL-24-e14462-s007.pdf]

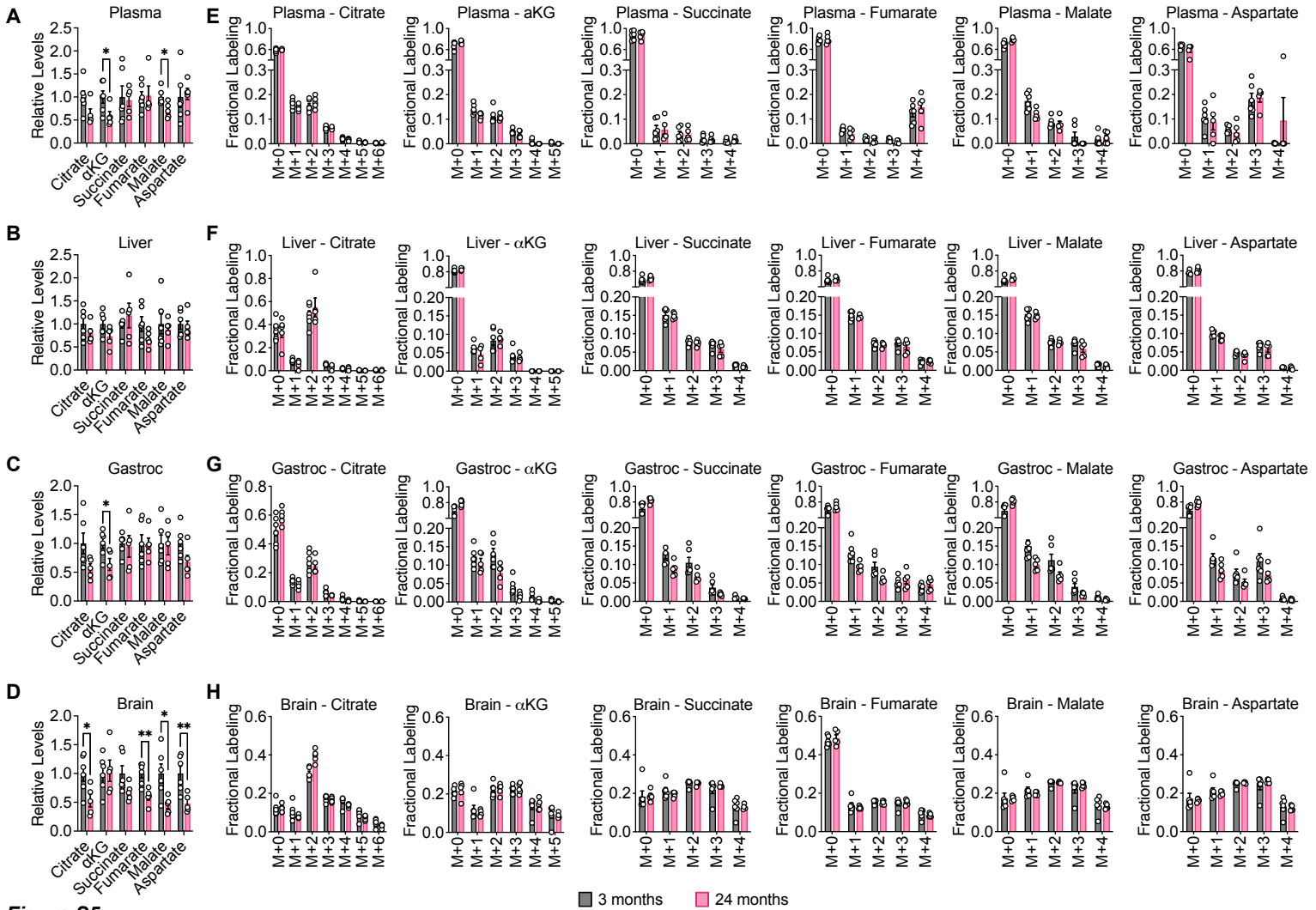

**Figure S5**

Supplement: Supplementary file 5 — Figure S5. Glucose contribution to the TCA cycle is robust in aging WSB/EiJ mice. WSB/EiJ mice, 3‐month‐old (n = 6) versus 24‐month‐old (n = 5), were infused with [U‐13C]‐glucose at 0.4 mg/min for 6 h. Relative levels of TCA cycle metabolites in plasma (A), liver (B), gastrocnemius muscle (C), and brain (D) tissues. Relative metabolite levels represent mass spectrometry peak areas that are normalized to an internal standard and tissue weight, before being normalized relative to the average value in 3‐month‐old mice. Mass isotopomer distributions of the indicated TCA cycle metabolites in plasma (E), liver (F), gastrocnemius muscle (G), and brain (H) tissues. Data are presented as mean ± SEM. Comparisons were made using a two‐tailed Student’s t test. *p< 0.05, **p< 0.01. [file ACEL-24-e14462-s018.pdf]

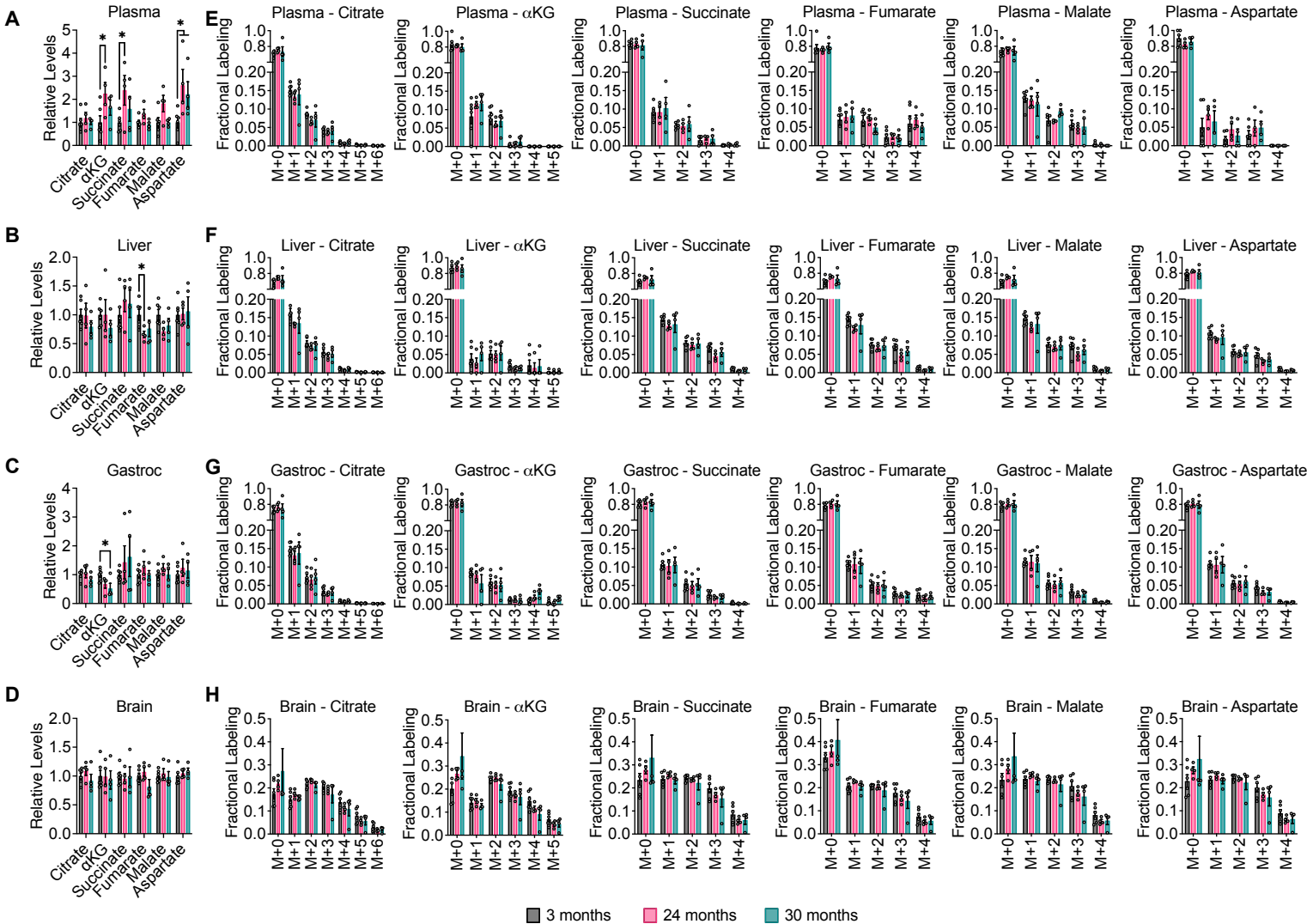

**Figure S6**

Supplement: Supplementary file 6 — Figure S6. Glucose contribution to the TCA cycle is robust in aging DO mice. DO mice, 3‐month‐old (n = 6), 24‐month‐old (n = 4), and 30‐month‐old (n = 4), were infused with [U‐13C]‐glucose at 0.4 mg/min for 6 h. Relative levels of TCA cycle metabolites in plasma (A), liver (B), gastrocnemius muscle (C), and brain (D) tissues. Relative metabolite levels represent mass spectrometry peak areas that are normalized to an internal standard and tissue weight, before being normalized relative to the average value in 3‐month‐old mice. Mass isotopomer distributions of the indicated TCA cycle metabolites in plasma (E), liver (F), gastrocnemius muscle (G), and brain (H) tissues. Data are presented as mean ± SEM. Comparisons were made using a two‐tailed Student’s t test. *p< 0.05. [file ACEL-24-e14462-s015.pdf]

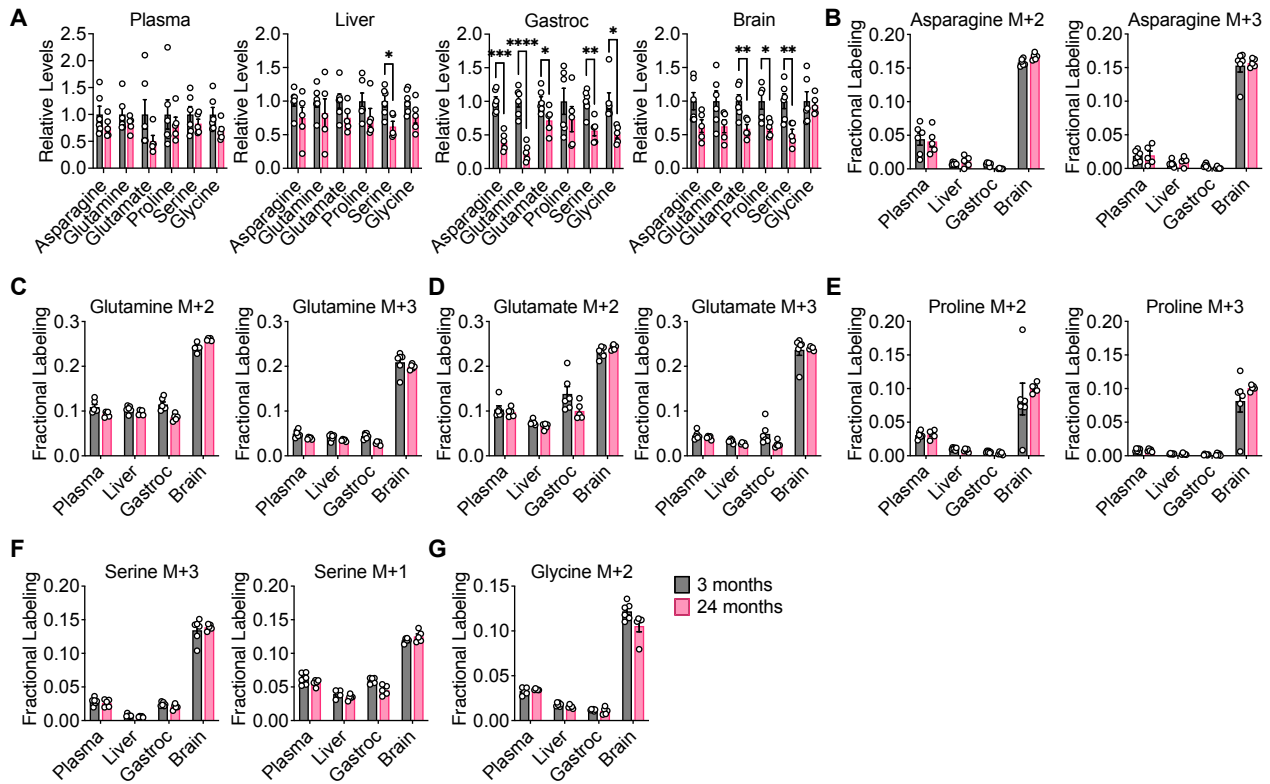

**Figure S7**

Supplement: Supplementary file 7 — Figure S7. Glucose contribution to amino acid metabolism is robust in aging WSB/EiJ mice. WSB/EiJ mice, 3‐month‐old (n = 6) versus 24‐month‐old (n = 5), were infused with [U‐13C]‐glucose at 0.4 mg/min for 6 h. (A) Relative levels of the indicated amino acids in plasma, liver, gastrocnemius muscle, and brain tissues. Relative metabolite levels represent mass spectrometry peak areas that are normalized to an internal standard and tissue weight, before being normalized relative to the average value in 3‐month‐old mice. Fractional labeling of [M + 2] and [M + 3] asparagine (B), [M + 2] and [M + 3] glutamine (C), [M + 2] and [M + 3] glutamate (D), [M + 2] and [M + 3] proline (E), [M + 3] and [M + 1] serine (F), and [M + 2] glycine (G) in the indicated tissues. Data are presented as mean ± SEM. Comparisons were made using a two‐tailed Student’s t test. *p< 0.05, **p< 0.01, ***p< 0.001, ****p< 0.0001. [file ACEL-24-e14462-s006.pdf]

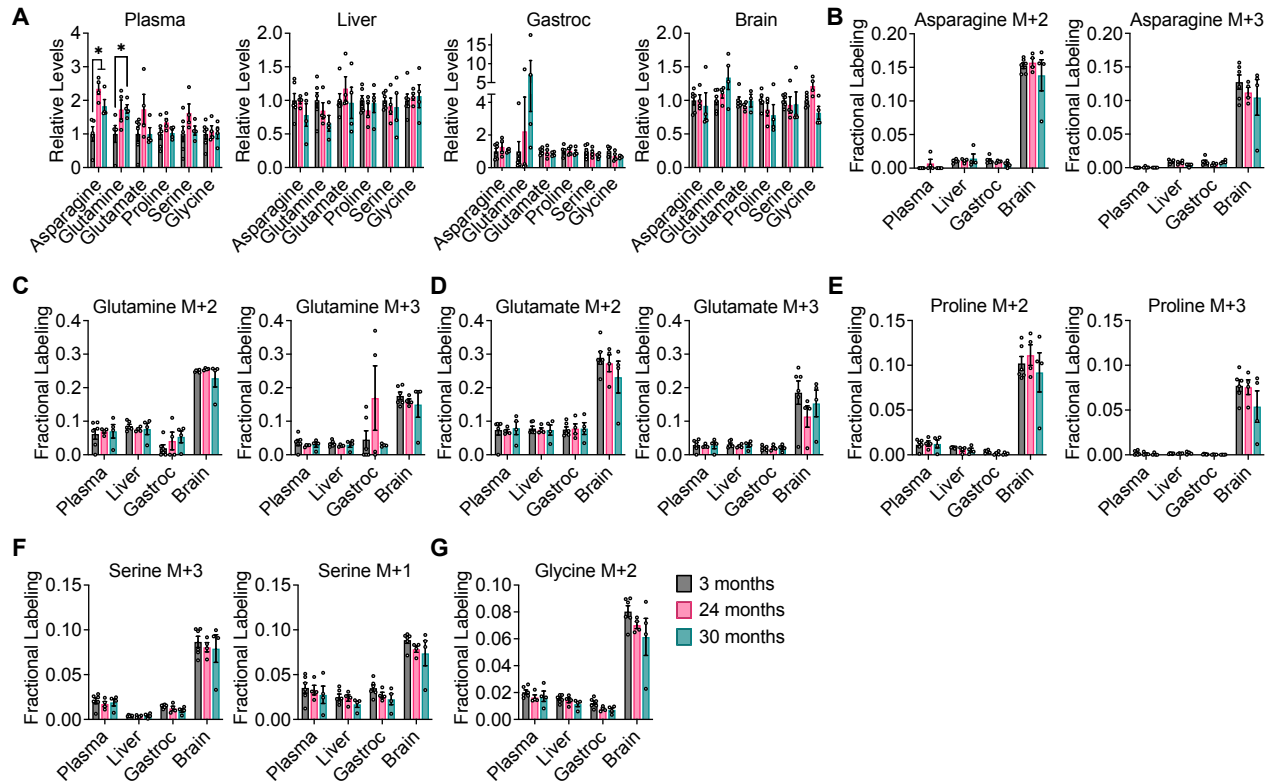

**Figure S8**

Supplement: Supplementary file 8 — Figure S8. Glucose contribution to amino acid metabolism is robust in aging DO mice. DO mice, 3‐month‐old (n = 6), 24‐month‐old (n = 4), and 30‐month‐old (n = 4), were infused with [U‐13C]‐glucose at 0.4 mg/min for 6 h. (A) Relative levels of the indicated amino acids in plasma, liver, gastrocnemius muscle, and brain tissues. Relative metabolite levels represent mass spectrometry peak areas that are normalized to an internal standard and tissue weight, before being normalized relative to the average value in 3‐month‐old mice. Fractional labeling of [M + 2] and [M + 3] asparagine (B), [M + 2] and [M + 3] glutamine (C), [M + 2] and [M + 3] glutamate (D), [M + 2] and [M + 3] proline (E), [M + 3] and [M + 1] serine (F), and [M + 2] glycine (G) in the indicated tissues. Data are presented as mean ± SEM. Comparisons were made using a two‐tailed Student’s t test. *p< 0.05. [file ACEL-24-e14462-s017.pdf]

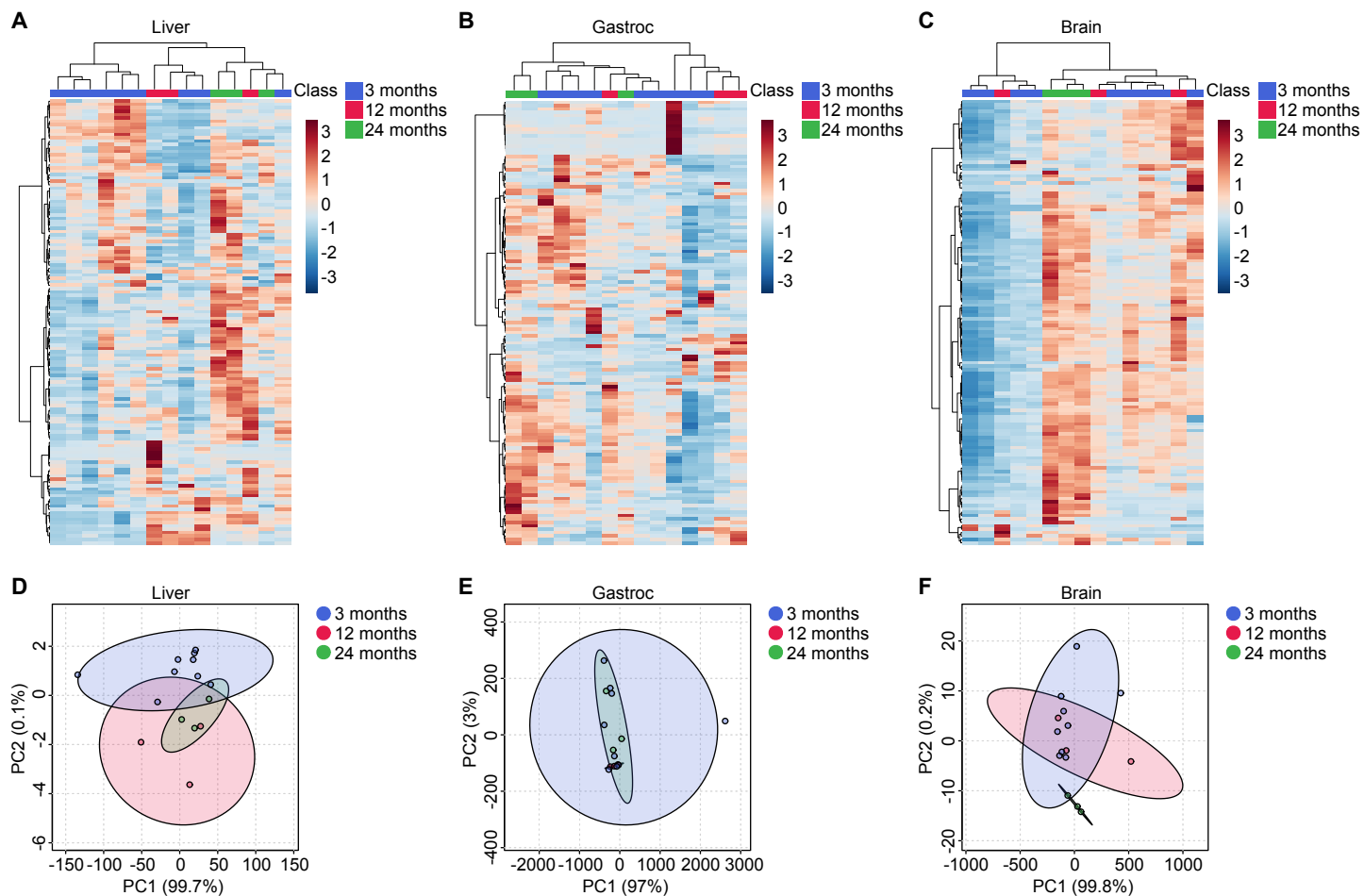

**Figure S9**

Supplement: Supplementary file 9 — Figure S9. Polar metabolite profiling does not reveal strong age‐dependent changes in metabolite levels. Polar metabolite levels were measured by LC–MS in tissues from uninfused 3‐month‐old (n = 9), 12‐month‐old (n = 3), and 24‐month‐old (n = 3) C57BL/6J mice. Data were analyzed using MetaboAnalyst. Heat maps of metabolite levels from liver (A), gastrocnemius muscle (B), and brain (C) tissues. Principal component analysis of metabolite levels from liver (D), gastrocnemius muscle (E), and brain (F) tissues. [file ACEL-24-e14462-s003.pdf]

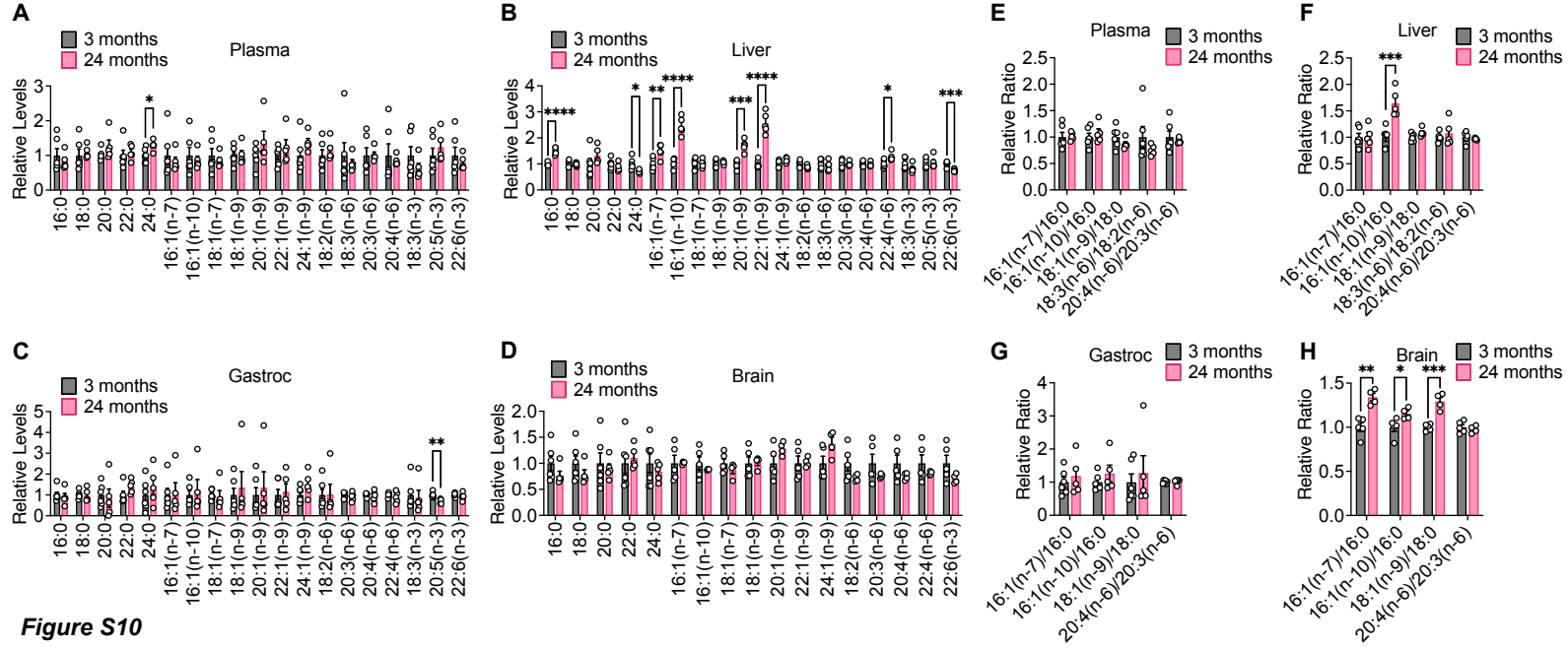

Supplement: Supplementary file 10 — Figure S10. Fatty acid desaturation increases in tissues from aging WSB/EiJ mice. Relative levels of the indicated fatty acids in plasma (A), liver (B), gastrocnemius muscle (C), and brain (D) tissues from young versus old WSB/EiJ mice. Relative fatty acid levels represent mass spectrometry peak areas that are normalized to an internal standard and tissue weight, before being normalized relative to the average value in 3‐month‐old mice. Relative fatty acid desaturation ratios in plasma (E), liver (F), gastrocnemius muscle (G), and brain (H) tissues from young versus old WSB/EiJ mice. Relative fatty acid ratios are shown normalized to the average value in 3‐month‐old mice. 3 months n = 6, 24 months n = 5. Data are presented as mean ± SEM. Comparisons were made using a two‐tailed Student’s t test. *p< 0.05, **p< 0.01, ***p< 0.001, ****p< 0.0001. [file ACEL-24-e14462-s002.pdf]

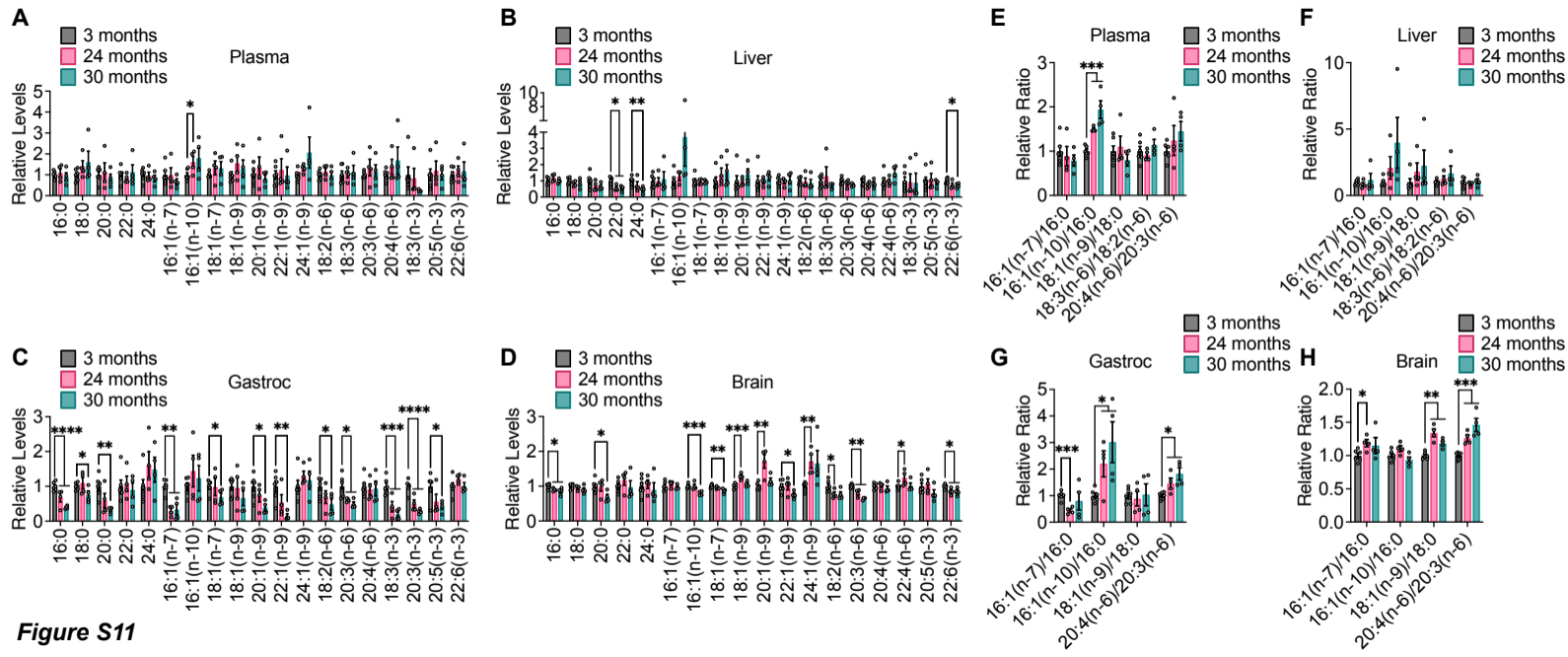

Supplement: Supplementary file 11 — Figure S11. Fatty acid desaturation increases in tissues from aging DO mice. Relative levels of the indicated fatty acids in plasma (A), liver (B), gastrocnemius muscle (C), and brain (D) tissues from young versus old DO mice. Relative fatty acid levels represent mass spectrometry peak areas that are normalized to an internal standard and tissue weight, before being normalized relative to the average value in 3‐month‐old mice. Relative fatty acid desaturation ratios in plasma (E), liver (F), gastrocnemius muscle (G), and brain (H) tissues from young versus old DO mice. Relative fatty acid ratios are shown normalized to the average value in 3‐month‐old mice. 3 months n = 6, 24 months n = 4, 30 months n = 4. Data are presented as mean ± SEM. Comparisons were made using a two‐tailed Student’s t test. *p< 0.05, **p< 0.01, ***p< 0.001, ****p< 0.0001. [file ACEL-24-e14462-s012.pdf]

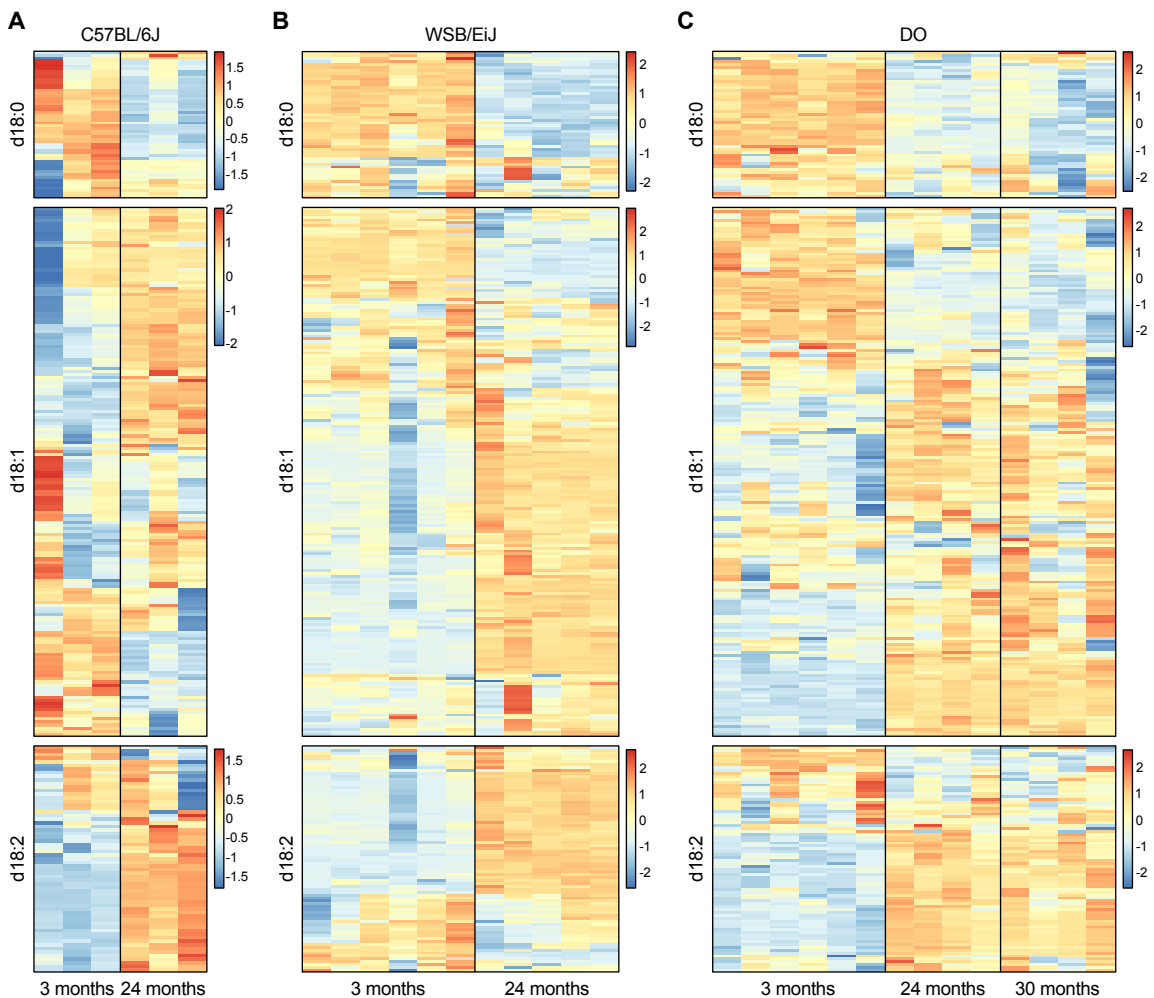

**Figure S12**

Supplement: Supplementary file 12 — Figure S12. Aging brain tissue exhibits changes in levels of d18:0‐, d18:1‐, and d18:2‐containing sphingolipid species. Heat maps of relative levels of d18:0‐, d18:1‐, and d18:2‐containing sphingolipid species in brain tissues from young versus old C57BL/6J (A), WSB/EiJ (B), and DO (C) mice. Heat map scale bars represent z scores. C57BL/6J: 3 months n = 3, 24 months n = 3. WSB/EiJ: 3 months n = 6, 24 months n = 5. DO: 3 months n = 6, 24 months n = 4, 30 months n = 4. [file ACEL-24-e14462-s001.pdf]

**A**

C57BL/6J

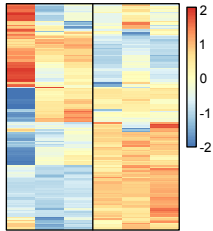

3 months 24 months

**B**

WSB/EiJ

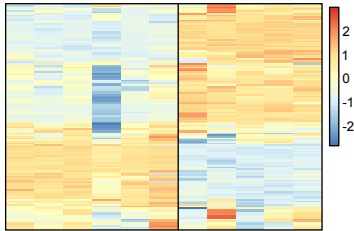

3 months

24 months

**C**

DO

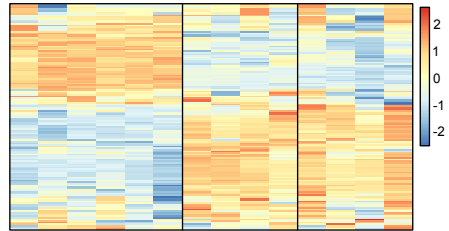

3 months

24 months

30 months

**Figure S13**

Supplement: Supplementary file 13 — Figure S13. Aging brain tissue exhibits changes in levels of sphingolipid species that contain 2‐hydroxylated fatty acids. Heat maps of relative levels of sphingolipid species that contain 2‐hydroxylated fatty acids in brain tissues from young versus old C57BL/6J (A), WSB/EiJ (B), and DO (C) mice. Heat map scale bars represent z scores. C57BL/6J: 3 months n = 3, 24 months n = 3. WSB/EiJ: 3 months n = 6, 24 months n = 5. DO: 3 months n = 6, 24 months n = 4, 30 months n = 4. [file ACEL-24-e14462-s008.pdf]
