# Supplementary material for: Effects of Aging on Glucose and Lipid Metabolism in Mice
Source: Aging Cell. 2024 Dec 27;24(4):e14462. doi: 10.1111/acel.14462 (PMC11984682; doi:10.1111/acel.14462)
Supplement: Supplementary file 14 — Table S1. Infusion rate parameters for optimizing [U‐13C]‐glucose infusions. [file ACEL-24-e14462-s014.docx]

**Table S1. Infusion rate parameters for optimizing [U-^13^C]-glucose infusions**

| **Mouse #** | **Body Weight (g)** | **[U-^13^C]-glucose Solution Concentration (mg/ml)** | **Pump Rate (µl/min)** | **Final Infusion Rate** |
| --- | --- | --- | --- | --- |
| 1 | 25.6 | 500 | 1.5 | 30 mg/kg/min |
| 2 | 26 | 250 | 1.5 | 15 mg/kg/min |
| 3 | 25.9 | 100 | 1.5 | 6 mg/kg/min |
| 4 | 22.5 | 500 | 0.8 | 0.4 mg/min |
| 5 | 23.4 | 250 | 0.8 | 0.2 mg/min |
