# Supplementary material for: Effects of Aging on Glucose and Lipid Metabolism in Mice
Source: Aging Cell. 2024 Dec 27;24(4):e14462. doi: 10.1111/acel.14462 (PMC11984682; doi:10.1111/acel.14462)
Supplement: Supplementary file 15 — Table S2. Body weights (g) of all infused mice. [file ACEL-24-e14462-s016.docx]

**Table S2. Body weights (g) of all infused mice**

| **C57BL/6J**  **3 months** | **C57BL/6J**  **24 months** | **WSB/EiJ**  **3 months** | **WSB/EiJ**  **24 months** | **DO**  **3 months** | **DO**  **24 months** | **DO**  **30 months** |
| --- | --- | --- | --- | --- | --- | --- |
| 25 | 33.8 | 14.7 | 22.5 | 32.5 | 53.9 | 28.1 |
| 24.4 | 34.2 | 17.7 | 23.8 | 29.5 | 43.3 | 30.7 |
| 24.6 | 30.6 | 18 | 23.8 | 22.6 | 34.6 | 36.9 |
| 27.2 | 31.3 | 15.5 | 24.3 | 23.3 | 37.5 | 40.1 |
|  |  | 18 | 22.4 | 25.7 |  |  |
|  |  | 14.7 |  | 37.1 |  |  |
